# Kneel, stand, prostrate: The psychology of prayer postures in three world religions

**DOI:** 10.1371/journal.pone.0306924

**Published:** 2024-08-22

**Authors:** Patty Van Cappellen, Megan E. Edwards, Shanmukh V. Kamble, Mualla Yildiz, Kevin L. Ladd

**Affiliations:** 1 Social Science Research Institute, Duke University, Durham, North Carolina, United States of America; 2 Department of Psychology, Karnatak University, Dharwad, India; 3 Faculty of Divinity, Ankara University, Ankara, Turkey; 4 Department of Psychology, Indiana University South Bend, South Bend, Indiana, United States of America; King Khalid University, EGYPT

## Abstract

Most people practice a religion, often multiple times daily. Among the most visible aspects of these practices are body postures, which according to embodiment theories, likely shape the psychological experience of religion. In a preregistered study, we test this idea among Christians, Muslims, and Hindus in the United States, Turkey, and India (*N* = 2,458). In a repeated-measures experimental design, participants imagined praying in various typical postures, then reported their affective experiences, perceived relationship with deity, and prayer content for each posture. Compared to downward and constrictive postures, expansive and upward postures led to more positive emotions, dominance, and praise-focused prayers, yet fewer introspective or intercessory prayers. Interestingly, these effects varied based on religious context (e.g., many Hindus found upward and expansive postures offensive, causing no positive affect). We further explored whether these effects varied based on posture familiarity, religiosity, interoceptive sensibility, and personality traits. This research provides unique data on embodied processes shaping affect and cognition in religious practices.

## Introduction

Religions have played an important role in human societies throughout history, providing a framework for meaning-making, community building, and personal growth. A significant portion of humanity—over 80% or 6.12 billion people in 2015—identified with a religion, with projections indicating a rise to 8.41 billion by 2060, predominantly driven by Christians, Muslims, and Hindus [1]. Moreover, across cultures and traditions, religions involve frequent practices such as daily prayer [2], which involve various sensory modalities including sound, sight, and touch, and often incorporate specific body postures and movements. Although the significance of these postures may differ across traditions, embodiment research in psychology suggests that they are not merely ornamental but may play a fundamental role in shaping the psychological experience of religious practices [3–6](_((((xxx. Shifting from religion as a mental construct, this research explores whether prayer postures influence emotions, perceived relationship with deity, and prayer content–across the three largest world religions–Christianity, Islam, and Hinduism.

According to an embodied framework, our cognitive processes, emotions, and actions are intimately connected to and influenced by the physical sensations and states of our body (for reviews see [7, 8]. Research in this domain highlights that our mental processes involve simulations of how we perceive and act in the world through our physical bodies. Activating one sensory modality (e.g., kinesthesia, the sense of movement), can trigger a network of interconnected and multimodal experiences, ranging from basic affect to semantic concepts [9]. The present research leverages *imagined* postures to test the causal effect of posture on experience since mentally imagining an action engages many of the same neural and cognitive processes involved in the actual performance of the action [10]. Consequently, imagined action is a point of entry to trigger the network of multimodal stored experiences and can trigger emotions and thoughts connected with the action [7, 11, 12](_((((xxx. This theory and accompanying research suggest that the postures worshippers adopt are far from inconsequential; rather, they serve as activations within an interconnected network of experiences.

To organize the many postures that worshippers can adopt, we focus on two dimensions identified in the broader embodiment literature: 1) upward-downward orientation; and 2) expansive-constrictive. Though various religions’ prayer postures comprise of different-looking postures, the most frequently adopted postures across faith traditions fall into three distinct families that encompass these two dimensions: 1) upward and expansive postures (e.g., looking upward and reaching up); 2) downward and constrictive postures (e.g., kneeling and bowing the head or full body); 3) resting postures (e.g., head straight, sitting or standing with arms relaxed) [6, 13](_((((xxx. For this research, we focus on the first two postural families, being most likely to elicit divergent feelings and thoughts.

To capture multiple facets of prayer experience, we study how prayer postures relate to emotions, perceived relationship with deity, and prayer content. For emotions, we investigate three established dimensions of subjective feeling–valence (pleasantness), arousal (activation), and dominance [PAD model, 14, see 15] –along with specific positive and negative emotions. Perceived relationship with deity was examined through closeness to deity, as well as motivations to move toward growth in (approach), and avoiding harming, the relationship with deity (avoidance [16]). For prayer content, prayers involve different communicative genres and emotional registers [17], resulting in several typologies of prayer. Adopting the same prayer typology as in (_((((xxxVan Cappellen and colleagues [18](_((((xxx, we have expanded and organized prayer content by valence (positive or negative) and the self-God-other people focus of the prayer [cf. 19].

Research in grounded cognition, affective science, and religion provides a foundation to articulate our expectations about the relationship between the two families of prayer postures and their associated emotions and religious experiences. On one hand, downward and constrictive postures are related to: negative affect [20, 21], feelings of helplessness [22], submission, and humility [23, 24]. On the other hand, upward and expansive postures are related to: positive affect [20, 25, 26](_((((xxx, dominance or pride [27–29] see review [30](_((((xxx. Moreover, adopting a typical upward and expansive *prayer* posture (i.e., standing with hands raised and looking up) led to more positive affect and more parasympathetic activity, indexed through respiratory sinus arrhythmia, than a typical downward and constrictive prayer posture (i.e., standing with head down and prayer hands) [31].

Beyond outcomes related to emotions, cross-sectional studies suggest that downward and constrictive prayer postures are associated with repentance and confession, both of which involve greater self-focus and more difficult emotions. In contrast, upward and expansive postures prayer are associated with praise, and to a lesser extent thanksgiving, which involve more positive emotions [18, 32].

Much of the cited research has overlooked the religious context in which these postures can be assumed and is limited by samples that are small and/or predominantly Christian and Western. In the present research, we increase religious culture representation and explore whether prayer postures activate similar networks of stored experiences. We consider prayer postures as functioning to coordinate the social interaction in the context of (nonverbal) communication with a deity. As religious traditions share similar broad conceptualizations of their deities (powerful, omnipotent, benevolent and authoritarian, deserving respect and devotion, and with the potential to be pleased or offended [33]), we expect that prayer postures will express and elicit similar emotions and cognitions across traditions.

Thus, the present investigation seeks to expand upon prior research in several significant ways: (a) larger samples, (b) representation of religiously and culturally diverse participants, (c) more directly testing the psychological effects of postures adopted in a religious context. Finally, we also extend such research by (d) exploring the role of individual differences as moderators of embodiment effects [6] and more specifically religiosity, personality, and how much one is attuned to their body sensations [34].

Overall, we hypothesized the following (preregistration at https://osf.io/2fxjg/; rephrased here for precision): Compared to downward and constrictive postures, imagined *upward and expansive* postures will lead to: greater positive emotions, arousal, and dominance (omitted by error from the preregistration); approach motivation toward deity; and positive-valence prayer content of praise and thanksgiving. However, compared to upward and expansive postures, imagined *downward and constrictive* postures will lead to: greater humility; closeness to deity; and negative-valence prayer content of examining one’s difficulties and intercession for others.

We hypothesized that these findings would replicate for each religion (Christianity, Islam, and Hinduism) of Sample A (U.S.), as well as in Samples B (Turkey) and C (India). Lastly, we planned to explore the moderating role of: experience with the postures, religiosity, and frequency of religious practices; and additionally in Sample A, interoceptive sensibility and Big Five personality traits.

This research was preregistered on Open Science Framework (preregistered codebook and recruitment materials: https://osf.io/s62zu; preregistration of hypotheses, data collection plan and analyses: https://osf.io/2fxjg; and the dataset and associated codebook are freely accessible: https://osf.io/hm7d5/).

## Method

### Participants: Sample A

Participants were recruited through Amazon’s Mechanical Turk and Turk Prime. Participants had to identify as Christian, Muslim, or Hindu and live in the U.S. in order to participate. The study was advertised as a 45-minute psychology survey about postures and emotions across religions and was individually targeted toward each religious group in order to achieve the desired sample size for each, preregistered as 750 Christians, 500 Muslims, and 500 Hindus. Participants were compensated $3.00 USD for completing the online survey. Participants who did not pass initial attention check questions or inclusion requirements were eliminated from participation and not compensated. After data collection was complete, out of 1,707 participants, 137 were excluded from analyses based on the following criteria: they finished the survey in less than five minutes, failed to follow directions to written response questions, and/or selected the same response option across an entire scale where such answer patterns would not make sense reverse-scored items or items measuring separate dimensions such as positive and negative emotions (with the exception of selecting a neutral or not at all scale option). The final set of participants (*N* = 1,570; Christian *n* = 674; Muslim *n* = 494; Hindu *n* = 402) was 47.1% female, 52.7% male, and .1% other. Age ranged from 18–81 years (*M*_*age*_ = 35.17, *SD*_*age*_ = 11.66). The majority of participants identified as Caucasian, 48.7%; 30.4% Asian, 11.2% African American, 4.5% other, 4.3% American Indian or Alaska Native, .7% Native Hawaiian or other Pacific Islander, and .2% did not respond.

### Participants: Sample B

Participants (*N* = 498) from Turkey were recruited from a University in Ankara. Participants needed to identify as Muslim and have grown up in Turkey. Participants that did not match these criteria were excluded, leaving a final sample of *N* = 452 (63.3% female, 4% did not respond; *M*_*Age*_ = 21.56, *SD*_*Age*_ = 4.65).

### Participants: Sample C

Participants (*N* = 500) were recruited from a University in Southern India. Participants needed to speak English, identify as Hindu, and have grown up in India. Participants that did not match these criteria were excluded, leaving a final sample size of *N* = 436 (62.2% female; *M*_*Age*_ = 22.64, *SD*_*Age*_ = 1.91).

### Procedure

Procedure and materials were approved by the Institutional Review Board at the institution of the first author. Participants in all samples gave consent electronically or via paper form prior to participating in the research. The study ran from February 2019 until October 2019. The present research employs imagined postures to test the causal effect of postures on experience. Practically, the use of imagined postures is the most effective method for collecting data on multiple postures in large samples, especially when compared to the alternative of physically adopting each posture within a laboratory setting. This method enables us to gather data beyond laboratory settings, thus granting us access to a more diverse population. We use a within-subject experimental design in which all participants are exposed to all manipulations (i.e., all postures). After each manipulation, participants completed the dependent variables.

**Fig 1:**
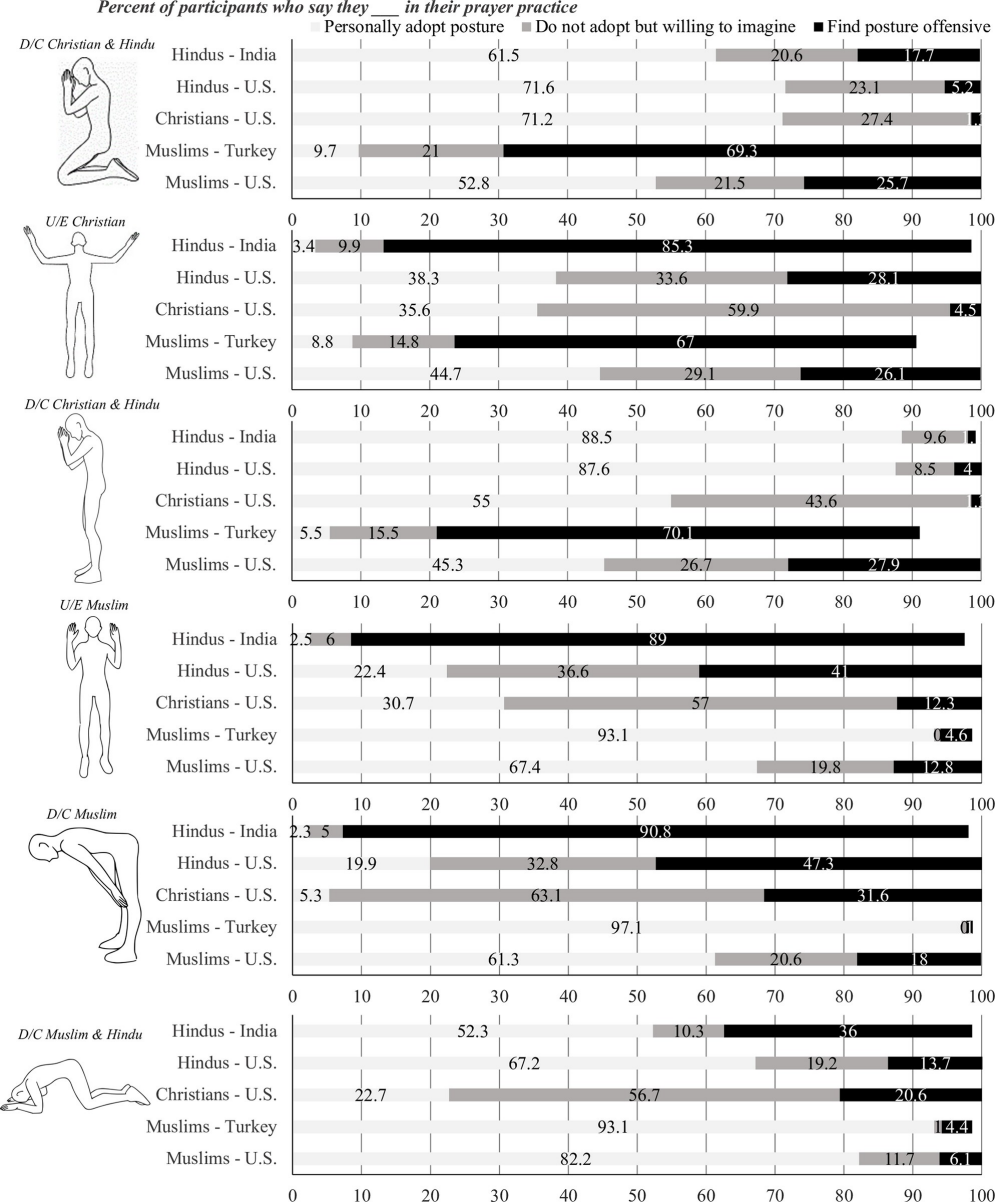
Frequencies of posture display rules for all samples. D/C is Down/Constrictive; U/E is Up/Expansive. Religious Tradition of the Posture is Indicated above each Posture.

Participants were presented with images of different postures that we internally categorized as either *downward and constrictive* (four postures) or *upward and expansive* (two postures), see Fig 1. We also varied the order of presentation of these images. We presented the images of postures most typical of the participants’ religious tradition first and then of the other religious traditions. Participants were simply presented with the image of the posture without additional information about its postural dimensions or tradition (for example, participants were not told that a posture was categorized by the research team as downward and constrictive, nor that a posture was more typical of the Muslim tradition). We also collected data for a neutral posture, i.e., standing with arms at sides, which we do not report as it was not part of the hypotheses. This posture was originally included to explore whether a posture relatively neutral on our postural dimensions of interest could serve as a control posture, but we found that it was strongly associated with the Muslim practice and could therefore not serve as a “neutral” control across religions. The selection of prayer postures for this research was based on interviews with members of each religion and the authors’ knowledge of religious practices. Participants were shown computer drawn images of the prayer postures to avoid indicating gender or race.

Postures were presented one at a time and participants were asked to: “Imagine yourself adopting the postures in your private prayer (you may make small adjustments to the posture if you feel it is necessary). Think about where you would be, what you would be saying, what you would think of, and what you would feel. As you answer the questions below each posture, please continue to imagine being in that posture and let it guide your responses. Note that there are no right or wrong responses here, how you feel is completely personal to you.”

Then, to validate the selection of postures and to grasp display rules in each religious tradition, we asked participants upon viewing the posture if they: (a) personally adopt this posture during prayer; (b) do not adopt this posture during prayer, but are willing to *imagine* themselves in the posture; or (c) find this posture offensive and are not willing to imagine themselves praying in this posture. Importantly, participants who felt it offensive were not required to complete measures for the posture and were skipped to the next one (see Fig 1 for frequencies of display rules, and S1 Text in S1 File, for discussion). For each non-offensive posture that participants imagined praying in, they completed a series of questions on the perception of their thoughts and feelings during their prayer. After completing all postures, participants responded to general questions on their religiosity and frequency of religious practices, demographics; and additionally in Sample A, interoceptive sensibility and personality.

Participants in the U.S. (Sample A) completed the study online, whereas participants in Turkey (Sample B) and India (Sample C) completed the study with pen and paper. All measures were translated into Turkish for Sample B using translation-back translation and review by an additional bilingual expert. The fourth author administered the study locally in Turkey and informed that Turkish was the language typically used for research in this student population. These college student participants were recruited on a volunteer basis (the norm for participating in research at this university). Participants in India completed the study in English and were compensated with academic supplies. The third author administered the study locally in India and informed that English was the language typically used for teaching and research in this student population. To reduce the length of the study, participants in Turkey and India were not administered the individual difference measures of interoceptive sensibility and Big Five personality traits.

### Measures

#### Emotion

*Specific emotions*. Participants reported the degree to which they think they would feel ten distinct emotions while praying in each posture. To reduce the number of individual items for each posture, nine emotions were selected from the modified Differential Emotions Scale [35] instead of the full 20. We selected emotions that were most likely associated with prayer. A **positive emotions** subscale (6-items: *awe*, *grateful*, *hopeful*, *inspired*, *love*, and *proud*) and a **negative emotions** subscale (3-items: *ashamed*, *guilty*, and *sad*) were computed by averaging their respective items. To measure **humility**, we added a tenth emotion, *humble*. Participants responded on a scale from 0 (*not at all*) to 4 (*extremely*). Reliability for each subscale across postures was excellent (positive emotions: U.S. *α* = .97; Turkey *α* = .96; India *α* = .94; negative emotions: U.S. *α* = .96; Turkey *α* = .85; India *α* = .94).

*Affective dimensions*. For every prayer posture, we measured participants’ overall felt emotional **valence**, **arousal**, and **dominance** each with one-item [36]. Participants were asked how they would generally feel: valence was rated on a scale from 1 (*happy*) to 9 (*unhappy*); arousal, from 1 (*stimulated*) to 9 (*relaxed*); and dominance, from 1 (*submissive*) to 9 (*dominant*). Valence and arousal were reversed scored (higher scores indicating greater happiness or arousal).

#### Relationship with deity

*Closeness to God*. Participants reported how close they would feel to God in each posture. Adapted from Sharp and Johnson [37], two circles were shown where one represented God and another represented the self. Participants were asked to select a set of two circles which varied in distance, from 1 = *most distance between circles* to 5 = *total overlap between circles*.

#### Approach and avoidance motivation toward deity

Two items from the Social Approach and Avoidance Motives Scale [38] were adapted to assess participants’ approach and avoidance motives in the context of their relationship with God. Participants responded to the following prompt: “Imagining adopting the posture and thinking about God, I would be trying to …” Measuring approach: “Move toward growth and development in my relationship with God.”; measuring avoidance: “Stay away from situations that could harm my relationship with God.” Participants responded on an 8-point Likert type scale, from 0 = *not at all true of me* to 7 = *very true of me*.

#### Prayer content

To assess prayer content per posture, participants reported the extent to which their prayer reflected six different themes on a 7-point scale from 0 = *not at all* to 6 = *completely*. Three sets of two themes each represented a self-, other-, and God-focus, and each set was further delineated by positive or negative valence. The six themes are as follows: (1) *thanksgiving of personal things* (positive, self-focus), (2) *thanksgiving for other things* (positive, other-focus), (3) *praise toward God* (positive, God-focus), (4) *examination of one’s difficulties* (negative, self-focus), (5) *intercession for others* (negative, other-focus), and an exploratory (6) *negative emotions toward God* (negative, God-focus). For each prayer theme, examples were provided: “Examination of one’s difficulties (confession, repentance, making a personal request).”

#### Individual differences

*Experience with the postures*. Participants reported their familiarity with the postures. Two items asked how often they adopt each posture in both (a) private and (b) public religious practice. Participants responded on a 4-point scale of *never*, *rarely*, *sometimes*, *often*. Scores were averaged for these two items and across postures for an overall index of experience.

*Religiosity*. Overall religiosity was assessed with a three-item measure [19]: “To what extent do you consider yourself a religious person?”, “To what extent is your stance on religious issues important to you?”, and “To what extent is it likely that other people may view you as a very religious individual?” Participants rated their agreement with each item on a scale from 0 (*not at all*) to 6 (*completely*). Reliability was as follows: U.S. *α* = .86; Turkey *α* = .75; India *α* = .53.

**Religious Practice** was measured using four items [39](_((((xxx. Two items tapped into public religious activity: “How often do you attend religious services?” on a scale from 0 (*never*) to 8 (*several times a week*) and “How often do you take part in the activities of a place of worship other than attending services?” on a scale from 0 (*never*) to 10 (*several times a day*). Two items tapped into private religious practices: “How often do you pray privately in places other than at a specific religiously oriented building?” and “How often do you pray with other people in places other than at a specific religiously oriented building?” both on a scale from 0 (*never*) to 7 (*more than once a day*). Participants responses to each item were z-scored and then averaged. Reliability was as follows: U.S. *α* = .82; Turkey *α* = .69; India *α* = .68.

*Interoceptive Sensibility (Sample A only)*. Interoceptive sensibility refers to the conscious perception, processing, and recognition of internal bodily sensations [34]. To assess interoceptive sensibility, we used the Multidimensional Assessment of Interoceptive Awareness scale (α = .93; [34]). In this 21-item scale, participants provided ratings on a scale of 1 (*never*) to 5 (*always*). Due to time constraints, we only assessed five of the eight (32 items) subscales, which tapped most closely into interoceptive sensibility: Noticing (3-items; e.g., “When I am tense I notice where the tension is located in my body”), Attention Regulation (7-items; e.g., “I can pay attention to my breath without being distracted by things happening around me”), Emotional Awareness (5-items; e.g., “I notice how my body changes when I am angry”), Body Listening (3-items; e.g., “I listen for information from my body about my emotional state”), and Trusting (3-items; e.g., “I trust my body sensations”).

*Big Five personality traits (Sample A only)*. To assess personality, we used the Ten-Item Personality Inventory [40]. Participants rated two items for each of the following personality traits on a 7-point Likert scale from 1 (*strongly disagree*) to 7 (*strongly agree*): Extraversion, Agreeableness, Conscientiousness, Emotional Stability, and Openness to Experience.

## Results

Frequencies at which participants personally adopt each of the presented prayer postures by religious traditions are reported in Fig 1 (see S1 Text in S1 File for discussion, not central to hypotheses testing). We analyzed the data by comparing the two postural families on our outcome variables, averaging across the four down/constrictive postures, and across the two up/expansive postures. We present these results in Samples A and B: first, the main effects averaged across religious traditions, and then the interaction with religious traditions. These are followed by testing for moderators in Samples A and B: experience with the postures; religiosity; religious practice; and interoceptive sensibility and Big Five personality traits in Sample A only. Lastly, we present exploratory results for Sample C, as low frequency prevented us from testing primary hypotheses of the up/expansive postural family.

### Sample A: Overall differences between down/constrictive and up/expansive postures

To test for overall differences between postural families (down/constrictive and up/expansive postures) on all outcomes, we ran a one-way repeated measures ANOVA for the entire sample. See Table 1 for corresponding means, standard deviations, and significance tests.

**Table 1:** Means (standard deviations) of down/constrictive and up/expansive postures (U.S. sample a across religious groups).

		Down/Constrictive *M(SD*)	Up/Expansive *M(SD)*	*F* (1, 1419)	*p*	*η*_*p*_^*2*^	
**Emotions**							
	Positive emotions	**2.31 (0.87)**	**2.42 (0.93)**	**32.36**	**< .001**		**.022**
	Negative emotions	**1.05 (0.97)**	**.90 (1.02)**	**63.54**	**< .001**		**.043**
	Valence	**5.63 (1.83)**	**6.12 (1.97)**	**96.08**	**< .001**		**.063**
	Arousal	**4.69 (1.79)**	**5.29 (2.10)**	**154.26**	**< .001**		**.098**
	Dominance	**3.82 (1.83)**	**4.48 (1.90)**	**225.96**	**< .001**		**.137**
	Humility	**2.81 (0.98)**	**2.50 (1.15)**	**131.69**	**< .001**		**.085**
**Relationship with the deity**							
	Closeness to God	**3.69 (0.92)**	**3.58 (1.08)**	**22.52**	**< .001**		**.016**
	Approach Motivation	**4.28 (1.19)**	**4.15 (1.43)**	**14.95**	**< .001**		**.010**
	Avoidance Motivation	**3.82 (1.57)**	**3.62 (1.76)**	**37.08**	**< .001**		**.025**
**Prayer Content**							
	Thanksgiving of personal things	4.06 (1.29)	4.07 (1.50)	.06	.812		0
	Thanksgiving for other things	4.00 (1.29)	4.01 (1.50)	.103	.748		0
	Praise toward God	**4.21 (1.26)**	**4.43 (1.39)**	**35.01**	**< .001**		**.024**
	Examination of one’s difficulties	**4.16 (1.28)**	**3.50 (1.64)**	**260.82**	**< .001**		**.155**
	Intercession for others	**4.15 (1.23)**	**3.82 (1.52)**	**80.62**	**< .001**		**.054**
	Negative emotions toward God	1.81 (1.87)	1.77 (1.97)	1.49	.223		.001

*Note*. Differences statistically significant at *p* < .05 are bolded.

In line with our hypotheses, there was a significant difference between postural families on all measures of emotion. Specifically, participants reported significantly greater positive emotions, positive valence (happiness), arousal (stimulated), and dominance in the up/expansive postures compared to the down/constrictive postures. Whereas participants reported significantly greater negative emotions and humility in the down/constrictive postures compared to the up/expansive postures.

Regarding one’s relationship with deity, participants reported feeling significantly closer to God in down/constrictive postures. Surprisingly, they also reported both heightened approach *and* avoidance motivation toward God. Regarding prayer content, participants reported they would be involved in more prayers examining one’s difficulties and intercession for others in the down/constrictive postures compared to the up/expansive postures. By contrast, they reported more prayers of praise toward God in the up/expansive postures than down/constrictive postures. Contrary to expectations, there were no differences between postural families on thanksgiving of personal things, thanksgiving for other things, or negative emotions toward God.

#### Sample A: Religious traditions as potential moderator

**Fig 2:**
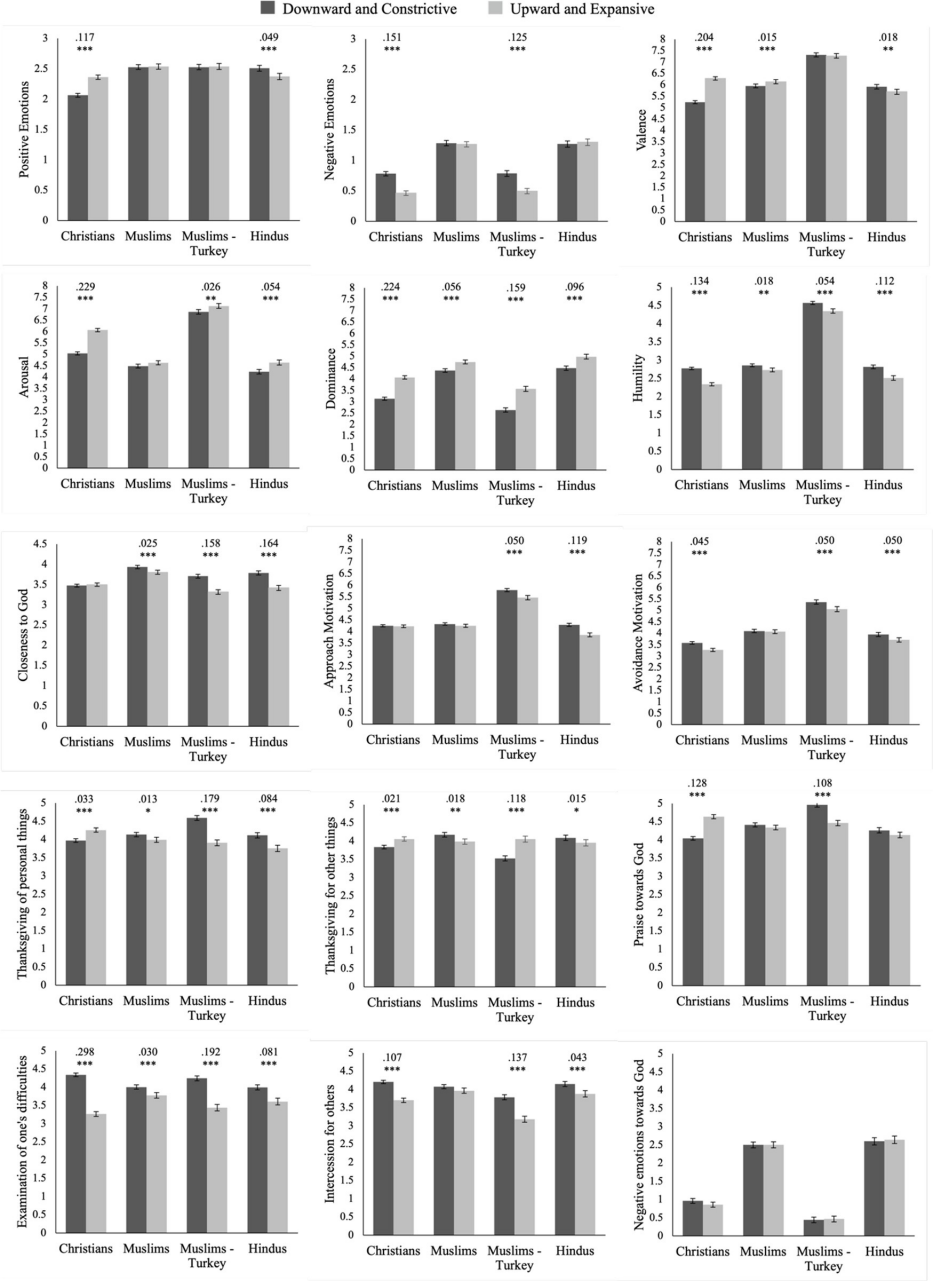
Means, standard errors, significance of difference, and effect sizes of down/constrictive and up/expansive postures by religious tradition from U.S. and Turkey (Samples A and B).

Using a two-way mixed ANOVA, we ran a 3 (Religious traditions: Christian vs. Muslim vs. Hindu) x 2 (Postural families: down/constrictive vs. up/expansive) analysis on all outcomes, where religious tradition was the between-subject factor and postural family was the within-subject factor, to test for interaction effects. For significant interactions, we followed up by testing simple main effects within each religious tradition. See Fig 2 for all means, standard errors, and statistical differences between conditions with effect size (see S2 Text in S1 File for written results with exact statistics).

Overall, Christians experienced more positive emotions and less negative emotions in up/expansive postures than in down/constrictive postures. Interestingly, Hindus showed the opposite pattern. Muslims, on the other hand, did not show differences in emotions between postural families, except in overall valence, which was similar to Christians. Participants from all religious traditions reported they would feel greater humility when imagining praying in down/constrictive postures, and greater dominance in up/expansive postures. For arousal, only Christians and Hindus reported greater arousal in up/expansive postures (noting that affective valence goes in opposite direction for these two groups).

Additionally, Muslims and Hindus reported they would feel closer to God when imagining praying in down/constrictive postures compared to up/expansive postures, whereas Christians did not report a difference between postural families. We found that Christians’ prayer content was more negative-valence (i.e., being in need/asking and confessing) in the down/constrictive postures, and more positive-valence (i.e., praise and thanksgiving) in the up/expansive postures, which aligned with their emotions. Interestingly, for Hindus we found that both positive- and negative-valence prayer content was greater in down/constrictive postures compared to up/expansive postures. Finally, Muslims only reported more positive-valence prayer content (thanksgiving for personal, other things) in the down/constrictive postures, but otherwise did not show distinct differences between prayer contents by posture family.

#### Sample B, Turkey: Differences between down/constrictive and up/expansive postures

We tested whether results for American Muslims in Sample A replicated among Turkish Muslims in Sample B. To test for differences between the down/constrictive and up/expansive postures amongst Turkish Muslims, we ran a repeated measures ANOVA (see Fig 2).

Overall, Turkish Muslims reported higher arousal, dominance, and less humility in the up/expansive postures than in the down/constrictive postures. They also reported fewer negative emotions in the up/expansive postures, but not more positive valence or positive emotions than in the down/constrictive postures. In the down/constrictive postures, Turkish Muslims reported stronger endorsement of all prayer content except thanksgiving for others, which was more strongly endorsed in the up/expansive postures. Finally, they reported feeling closer to God in the down/constrictive postures than in the up/expansive postures.

In follow-up analyses, we tested whether country (i.e., U.S. or Turkey) moderated the differences between the down/constrictive and up/expansive postures on experience (see S3 Text in S1 File). We found that, with the exception of valence and humility, when a difference between the two postural families on experience was present, it was always more marked for Muslims in Turkey than for Muslims in the U.S.

#### Samples A and B: Experience with the postures as a potential moderator

**Fig 3:**
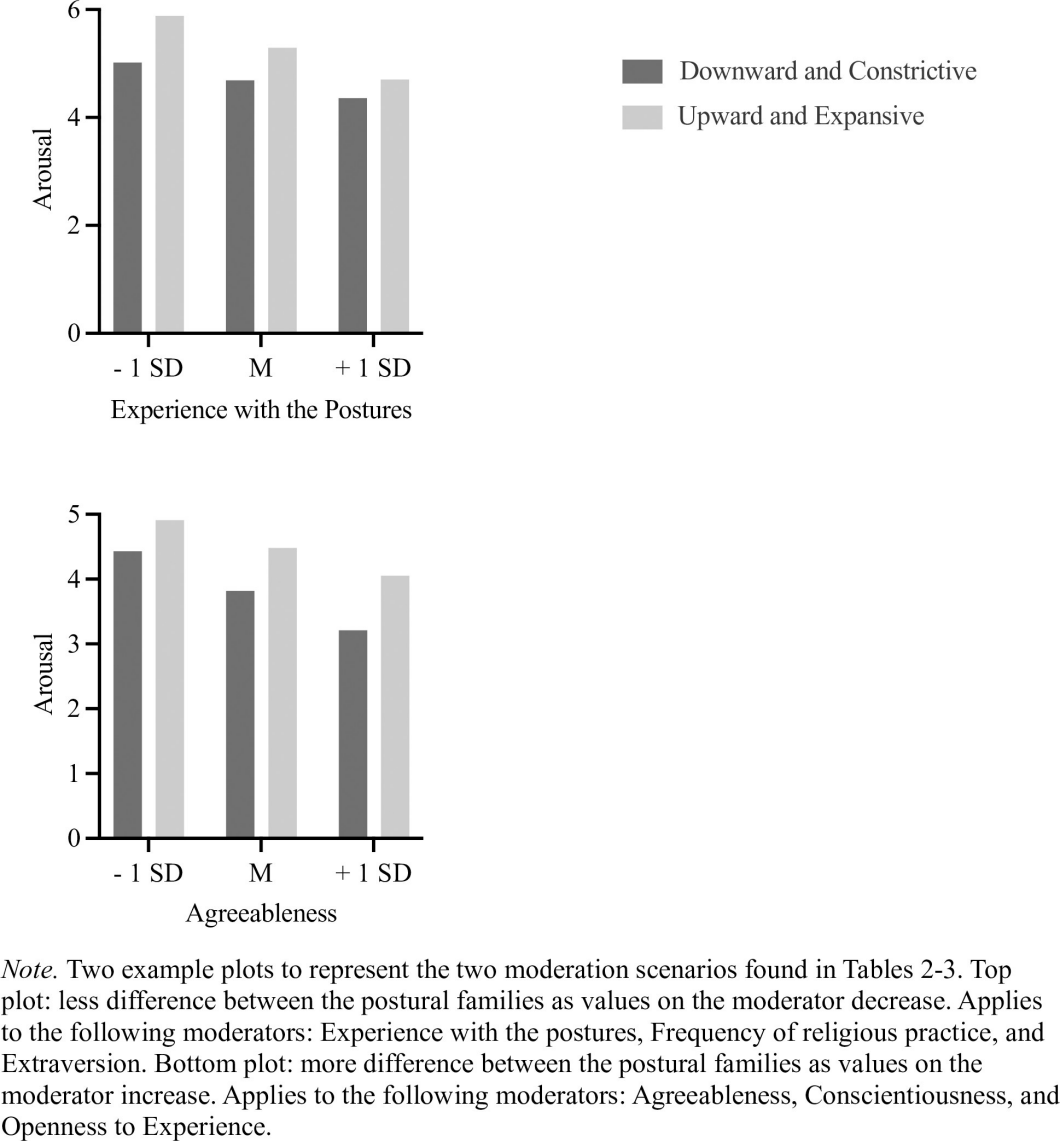
Moderations of postural families effects on experience by individual differences: Two scenarios. *Note*. Two example plots to represent the two moderation scenarios found in Tables 2 and 3. Top plot: less difference between the postural families as values on the moderator decrease. Applies to the following moderators: Experience with the postures, Frequency of religious practice, and Extraversion. Bottom plot: more difference between the postural families as values on the moderator increase. Applies to the following moderators: Agreeableness, Conscientiousness, and Openness to Experience.

To test whether experience with the postures would moderate the difference between the two postural families on the measured outcomes, we used the MEMORE v. 2.0 macro [41], which allows testing for moderation in a within-subject design using a continuous between-subject measure (i.e., experience). See Table 2 for tests of significance in Sample A (U.S.) and Fig 3 for plots. Experience with the postures was a significant moderator for all emotion outcomes (positive and negative emotions, valence, arousal, dominance, and humility), such that less experience was always associated with greater differences between down/constrictive and up/expansive postures. Similarly, experience was a significant moderator for prayer content of thanksgiving of personal things, praise toward God, examination of one’s difficulties, and intercession for others, where less experience was again associated with greater effects. However, experience was not a significant moderator for closeness to God, or the prayer content of thanksgiving for other things and negative emotions towards God. Results for Sample B (Turkish Muslim) can be found in S1 Table in S1 File. The pattern of results is similar such that whenever a significant interaction emerged (9/13 tests), more experience was associated with smaller differences between the two postural families on the measured outcomes.

**Table 2:** Moderation tests for difference between down/constrictive and up/expansive by experience with the postures and frequency of religious practice (U.S. Sample A across religious groups).

	Experience with the Postures			Frequency of Rel. Practice		
	*B*	*SE*	*95% CI*	*B*	*SE*	*95% CI*
**Emotions**						
Positive Emotions						
*Interaction*	.09***	.03	[.04, .14]	.06*	.03	[.01, .11]
*-1 SD*	-.18***	.03	[-.24, -.13]	.-16***	.03	[-.21, -.10]
*Mean*	-.11***	.02	[-.15, -.07]	-.11***	.02	[-.15, -.07]
*+1 SD*	-.04	.03	[-.10, .01]	-.07*	.03	[-.12, -.01]
Negative Emotions						
*Interaction*	-.08***	.02	[-.13, -.04]	-.08***	.02	[-.13, -.04]
*-1 SD*	.21***	.03	[.16, .26]	.21***	.03	[.16, .26]
*Mean*	.15***	.02	[.11, .18]	.15***	.02	[.11, .18]
*+1 SD*	.08**	.03	[.03, .13]	.09***	.03	[.03, .14]
Valence						
*Interaction*	.23***	.06	[.11, .35]	-.14*	.06	[-.27, -.02]
*-1 SD*	-.66***	.07	[-.79, -.52]	.59***	.07	[.45, .72]
*Mean*	-.48***	.05	[-.57, -.38]	.48***	.05	[.38, .57]
*+1 SD*	-.30***	.07	[-.43, -.17]	.37***	.07	[.23, .50]
Arousal						
*Interaction*	.34***	.06	[.21, .46]	-.20***	.06	[-.33, -.08]
*-1 SD*	-.87***	.07	[-1.00, -.74]	.76***	.07	[.63, .90]
*Mean*	-.61***	.05	[-.70, -.51]	.61***	.05	[.51, .70]
*+1 SD*	-.35***	.07	[-.48, -.21]	.45***	.07	[.32, .59]
Humility						
*Interaction*	-.31***	.03	[-.38, -.25]	-.22***	.03	[-.28, -.15]
*-1 SD*	.55***	.04	[.48, .62]	.47***	.04	[.40, .55]
*Mean*	.31***	.03	[.26, .36]	.31***	.03	[.26, .36]
*+1 SD*	.06	.04	[-.01, .14]	.14***	.04	[.07, .21]
Dominance						
*Interaction*	.53***	.06	[.42, .64]	.33***	.06	[.22, .44]
*-1 SD*	-1.07***	.06	[-1.19, -.96]	-.92***	.06	[-1.04, -.80]
*Mean*	-.66***	.04	[-.75, -.58]	-.66***	.04	[-.75, -.58]
*+1 SD*	-.26***	.06	[-.37, -.14]	-.41***	.06	[-.53, -.29]
**Relationship with the deity**						
Closeness to God						
*Interaction*	-.04	.03	[-.09, .03]	-.01	.03	[-.07, .05]
*-1 SD*	-	-	-	-	-	-
*Mean*	-	-	-	-	-	-
*+1 SD*	-	-	-	-	-	-
Approach Motivation						
*Interaction*	-.09*	.04	[-.17, -.01]	-.04	.04	[-.12, .05]
*-1 SD*	.20***	.05	[.11, .29]	-	-	-
*Mean*	.13***	.03	[.06, .19]	-	-	-
*+1 SD*	.06	.05	[-.03, .15]	-	-	-
Avoidance Motivation						
*Interaction*	-.20***	.04	[-.28, -.11]	-.13**	.04	[-.21, -.05]
*-1 SD*	.35***	.05	[.26, .44]	.30***	.05	[.21, .39]
*Mean*	.20***	.03	[.14, .26]	.20***	.03	[.14, .27]
*+1 SD*	.05	.05	[-.04, .14]	.10*	.05	[.01, .19]
**Prayer Content**						
Thanksgiving of personal things						
*Interaction*	.10*	.05	[.01, .19]	-.02	.05	[-.11, .08]
*-1 SD*	-.09	.05	[-.19, .02]	-	-	-
*Mean*	-.01	.04	[-.08, .06]	-	-	-
*+1 SD*	.07	.05	[-.03, .17]	-	-	-
Thanksgiving for other things						
*Interaction*	.06	.05	[-.03, .15]	-.01	.05	[-.11, .09]
*-1 SD*	-	-	-	-	-	-
*Mean*	-	-	-	-	-	-
*+1 SD*	-	-	-	-	-	-
Praise toward God						
*Interaction*	.22***	.05	[.18, .32]	.12*	.05	[.02, .21]
*-1 SD*	-.40***	.05	[-.50, -.29]	-.31***	.05	[-.42, -.21]
*Mean*	-.23***	.04	[-.30, -.15]	-.22***	.04	[-.30, -.15]
*+1 SD*	-.05	.05	[-.16, .05]	-.14*	.05	[-.24, -.03]
Examination of one’s difficulties						
*Interaction*	-.55***	.05	[-.65, -.45]	-.36***	.05	[-.47, -.26]
*-1 SD*	1.08***	.06	[.98, 1.19]	.94***	.06	[.83, 1.05]
*Mean*	.66***	.04	[.58, .73]	.66***	.04	[.58, .73]
*+1 SD*	.23***	.06	[.12, .34]	.37***	.06	[.26, .49]
Intercession for others						
*Interaction*	-.32***	.05	[-.41, -.23]	-.19***	.05	[-.28, -.10]
*-1 SD*	.58***	.05	[.48, .68]	.47***	.05	[.37, .57]
*Mean*	.33***	.04	[.26, .40]	.33***	.04	[.26, .40]
*+1 SD*	.08	.05	[-.02, .17]	.18***	.05	[.08, .28]
Negative emotions toward God						
*Interaction*	.04	.04	[-.04, .11]	.05	.04	[-.03, .13]
*-1 SD*	-	-	-	-	-	-
*Mean*	-	-	-	-	-	-
*+1 SD*	-	-	-	-	-	-

Note

***p < .001

** *p* < .01

* *p* < .05

#### Samples A and B: Religious practice as a potential moderator

Religious practice was tested as a potential moderator. In Sample A (U.S., see Table 2), religious practice was a significant moderator for all emotion outcomes (positive and negative emotions, valence, arousal, dominance, and humility) as well as for the prayer content of praise toward God, examination of one’s difficulties, and intercession for others. Again, greater religious practice was associated with smaller differences between the two postural families. However, the model was not significant for closeness to God, or the prayer content of thanksgiving for personal things, other things, and negative emotions towards God.

Surprisingly, in Sample B (Turkish Muslim), we found evidence of moderation on only one outcome: the prayer content of praise toward God, *B* = -.27, *SE* = .11, *t* = -2.50, *p* = .01, 95% CI [-.48, -.06]. Frequency of religious practice was associated with smaller differences between the two postural families (-1*SD*: *B* = .69, *SE* = .10, 95% CI [.49, .89]; *M*: *B* = .51, *SE* = .07, 95% CI [.37, .65]; +1*SD*: *B* = .33, *SE* = .10, 95% CI [.13, .53]).

#### Samples A and B: Religiosity as a potential moderator

We tested religiosity as a moderator but only found evidence of moderation on two emotion outcomes in Sample A (U.S.): dominance, *B* = .13, *SE* = .03, *t* = 3.72, *p* < .001, 95% CI [.06, .19], and humility, *B* = -.07, *SE* = .02, *t* = -3.32, *p* < .001, 95% CI [-.11, -.03]. Here, religiosity was associated with smaller differences between the two postural families for dominance (-1*SD*: *B* = -.83, *SE* = .06, 95% CI [-.95, -.71]; *M*: *B* = -.66, *SE* = .04, 95% CI [-.75, -.58; +1*SD*: *B* = -.50, *SE* = .06, 95% CI [-.62, -.38]) and humility (-1*SD*: *B* = .40, *SE* = .04, 95% CI [.32, .47]; *M*: *B* = .31, *SE* = .03, 95% CI [.26, .36]; +1*SD*: *B* = .22, *SE* = .04, 95% CI [.15, .29]).

In Sample B (Turkish Muslim), we found evidence of moderation on only one relationship with deity outcome: closeness to God, *B* = -.10, *SE* = .04, *t* = -2.22, *p* = .03, 95% CI [-.18, -.01]. Here, religiosity was associated with smaller differences the two postural families for closeness to God (-1*SD*: *B* = .51, *SE* = .07, 95% CI [.38, .64]; *M*: *B* = .40, *SE* = .05, 95% CI [.31, .50]; +1*SD*: *B* = .30, *SE* = .07, 95% CI [.17, .43]).

#### Sample A: Interoceptive sensibility as a potential moderator

We tested interoceptive sensibility as a moderator in Sample A (U.S.), and found evidence of moderation on two outcomes: humility, *B* = -.10, *SE* = .04, *t* = -2.57, *p* = .010, 95% CI [-.18, -.02], and the prayer content of negative emotions toward God, *B* = -.10, *SE* = .05, *t* = -2.21, *p* = .027, 95% CI [-.19, -.01]. Here, *lower* interoceptive sensibility was associated with greater differences the two postural families for both humility (-1*SD*: *B* = .38, *SE* = .04, 95% CI [.30, .45]; *M*: *B* = .31, *SE* = .03, 95% CI [.26, .36]; +1*SD*: *B* = .24, *SE* = .04, 95% CI [.16, .31]) and negative emotions toward God (-1*SD*: *B* = .11, *SE* = .04, 95% CI [.02, .19]; *M*: *B* = .04, *SE* = .03, 95% CI [0.03, .10]; +1*SD*: *B* = -.03, *SE* = .04, 95% CI [-.12, .05]). No other outcome variables were significantly moderated by interoceptive sensibility.

#### Sample A: Big Five personality traits as potential moderators

Finally, we tested each of the five personality traits as moderators in Sample A (U.S.) and found support for all except Emotional Stability. See Table 3 for all results. Extraversion consistently moderated the differences between the two postural families, making such differences *smaller* as Extraversion increased. Agreeableness and Conscientiousness also consistently moderated the effects, but differences between the two postural families became *larger* as these traits increased. Openness to Experience was a less consistent moderator (6/13 tests), but followed the same pattern as Agreeableness and Conscientiousness.

**Table 3:** Moderation tests for difference between down/constrictive and up/expansive by personality traits (U.S. Sample A across religious groups).

	Extraversion			Agreeableness			Conscientiousness			Openness to Experience		
	*B*	*SE*	*95% CI*	*B*	*SE*	*95% CI*	*B*	*SE*	*95% CI*	*B*	*SE*	*95% CI*
***Emotion***												
**Positive Emotions**												
*Interaction*	.03*	.01	[.0001, .06]	-.07***	.02	[-.10, -.04]	-.06***	.02	[-.09, -.03]	-.03*	.02	[-.07, -.003]
*-1 SD*	-.15***	.03	[-.20, -.10]	-.03	.03	[-.08, .03]	-.03	.03	[-.09, .02]	-.07*	.03	[-.12, -.02]
*Mean*	-.11***	.02	[-.15, -.07]	-.11***	.02	[-.15, -.07]	-.11***	.02	[-.15, -.07]	-.11***	.02	[-.15, -.07]
*+1 SD*	-.07**	.03	[-.13, -.02]	-.20***	.03	[-.25, -.14]	-.19***	.03	[-.24, -.14]	-.15***	.03	[-.21, -.10]
**Negative Emotions**												
*Interaction*	-.03*	.01	[-.06, -.004]	.07***	.02	[.04, .10]	.05***	.01	[.03, .08]	.05**	.02	[.02, .08]
*-1 SD*	.19***	.03	[.13, .24]	.06*	.03	[.01, .11]	.08**	.03	[.03, .13]	.09***	.03	[.04, .14]
*Mean*	.15***	.02	[.11, .18]	.15***	.02	[.11, .18]	.15***	.02	[.11, .18]	.15***	.02	[.11, .18]
*+1 SD*	.11***	.03	[.05, .16]	.24***	.03	[.18, .29]	.22***	.03	[.17, .27]	.21***	.03	[.15, .26]
**Valence**												
*Interaction*	.08*	.04	[.01, .15]	-.19***	.04	[-.27, -.11]	-.11**	.04	[-.18, -.04]	-.07	.04	[-.14, .01]
*-1 SD*	-.59***	.07	[-.73, -.46]	-.24***	.07	[-.37, -.11]	-.34***	.07	[-.47, -.20]	-	-	-
*Mean*	-.48***	.05	[-.57, -.38]	-.48***	.05	[-.57, -.38]	-.48***	.05	[-.57, -.38]	-	-	-
*+1 SD*	-.36***	.07	[-.50, -.23]	-.72***	.07	[-.85, -.58]	-.62***	.07	[-.75, -.48]	-	-	-
**Arousal**												
*Interaction*	.11**	.04	[.05, .18]	-.23***	.04	[-.31, -.16]	-.20***	.04	[-.27, -.12]	-.07	.04	[-.15, .004]
*-1 SD*	-.77***	.07	[-.90, -.63]	-.31***	.07	[-.45, -.18]	-.35***	.07	[-.49, -.22]	-	-	-
*Mean*	-.61***	.05	[-.70, -.51]	-.61***	.05	[-.70, -.51]	-.61***	.05	[-.70, -.51]	-	-	-
*+1 SD*	-.45***	.07	[-.58, -.31]	-.90***	.07	[-1.04, -.77]	-.86***	.07	[-1.00, -.73]	-	-	-
**Dominance**												
*Interaction*	.07*	.03	[.01, .13]	-.14***	.04	[-.21, -.07]	-.16***	.03	[-.23, -.09]	-.10**	.04	[-.17, -.03]
*-1 SD*	-.77***	.06	[-.89, -.64]	-.49***	.06	[-.61, -.37]	-.45***	.06	[-.58, -.34]	-.54***	.06	[-.66, -.41]
*Mean*	-.67***	.04	[-.75, -.58]	-.64***	.04	[-.75, -.58]	-.66***	.04	[-.75, -.58]	-.66***	.04	[-.75, -.58]
*+1 SD*	-.57***	.06	[-.69, -.44]	-.84***	.06	[-.96, -.72]	-.87***	.06	[-.99, -.75]	-.79***	.06	[-.92, -.67]
**Humility**												
*Interaction*	-.07***	.02	[-.10, -.03]	.06**	.02	[.02, .10]	.08***	.02	[.04, .12]	.04	.02	[-.01, .08]
*-1 SD*	.40***	.04	[.33, .47]	.23***	.04	[.16, .31]	.21***	.04	[.13, .28]	-	-	-
*Mean*	.31***	.03	[.26, .36]	.31***	.03	[.26, .36]	.31***	.03	[.26, .36]	-	-	-
*+1 SD*	.21***	.04	[.14, .29]	.38***	.04	[.31, .46]	.41***	.04	[.34, .48]	-	-	-
***Relationship with the Deity***												
**Closeness to God**												
*Interaction*	-.01	.02	[-.03, .04]	-.03	.02	[-.07, .003]	-.02	.02	[-.06, .01]	.001	.02	[-.04, .04]
*-1 SD*	-	-	-	-	-	-	-	-	-	-	-	-
*Mean*	-	-	-	-	-	-	-	-	-	-	-	-
*+1 SD*	-	-	-	-	-	-	-	-	-	-	-	-
**Approach Motivation**												
*Interaction*	.00	.02	[-.05, .05]	-.05	.03	[-.10, .01]	.01	.03	[-.04, .06]	-.01	.03	[-.07, .04]
*-1 SD*	-	-	-	-	-	-	-	-	-	-	-	-
*Mean*	-	-	-	-	-	-	-	-	-	-	-	-
*+1 SD*	-	-	-	-	-	-	-	-	-	-	-	-
**Avoidance Motivation**												
*Interaction*	-.07**	.02	[-.11, -.02]	.03	.03	[-.03, .08]	.04	.02	[-.01, .08]	-.01	.03	[-.06, .05]
*-1 SD*	.30***	.05	[.21, .39]	-	-	-	-	-	-	-	-	-
*Mean*	.20***	.03	[.14, .27]	-	-	-	-	-	-	-	-	-
*+1 SD*	.11*	.05	[.01, .20]	-	-	-	-	-	-	-	-	-
***Prayer Content***												
**Thanksgiving of personal things**												
*Interaction*	.07*	.03	[.01, .12]	-.08**	.03	[-.14, -.03]	-.05	.03	[-.11, .004]	-.01	.03	[-.07, .05]
*-1 SD*	-.10	.05	[-.20, .003]	.10	.05	[-.01, .20]	-	-	-	-	-	-
*Mean*	-.01	.04	[-.08, .06]	-.01	.04	[-.08, .06]	-	-	-	-	-	-
*+1 SD*	.08	.05	[-.02, .19]	-.11*	.05	[-.22, -.01]	-	-	-	-	-	-
**Thanksgiving for other things**												
*Interaction*	.03	.03	[-.03, .08]	-.07*	.03	[-.13, -.01]	-.03	.03	[-.09, .03]	-.02	.03	[-.08, .04]
*-1 SD*	-	-	-	.08	.05	[-.03, .18]	-	-	-	-	-	-
*Mean*	-	-	-	-.01	.04	[-.09, .06]	-	-	-	-	-	-
*+1 SD*	-	-	-	-.10	.05	[-.21, .001]	-	-	-	-	-	-
**Praise toward God**												
*Interaction*	.11***	.03	[.06, .16]	-.09**	.03	[-.15, -.03]	-.08**	.03	[-.13, -.02]	-.01	.03	[-.05, .07]
*-1 SD*	-.38***	.05	[-.48, -.27]	-.12*	.05	[-.22, -.01]	-.13*	.05	[-.23, -.02]	-	-	-
*Mean*	-.23***	.04	[-.30, -.15]	-.23***	.04	[-.30, -.15]	-.23***	.04	[-.30, -.15]	-	-	-
*+1 SD*	-.07	.05	[-.18, .03]	-.33***	.05	[-.44, -.23]	-.32***	.05	[-.43, -.22]	-	-	-
**Examination of one’s difficulties**												
*Interaction*	-.15***	.03	[-.21, -.10]	.20***	.03	[.14, .26]	.21***	.03	[.15, .27]	.11***	.03	[.05, .17]
*-1 SD*	.87***	.06	[.76, .98]	.40***	.06	[.29, .52]	.38***	.06	[.27, .49]	.52***	.06	[.41, .63]
*Mean*	.66***	.04	[.58, .74]	.66***	.04	[.58, .73]	.66***	.04	[.58, .73]	.66***	.04	[.58, .74]
*+1 SD*	.45***	.06	[.33, .56]	.91***	.06	[.80, 1.02]	.93***	.06	[.82, 1.05]	.79***	.06	[.68, .91]
**Intercession for others**												
*Interaction*	-.11***	.03	[-.16, -.06]	.12***	.03	[.06, .18]	.15***	.03	[.09, .20]	.12***	.03	[.06, .178]
*-1 SD*	.48***	.05	[.38, .58]	.18***	.05	[.08, .28]	.14**	.05	[.04, .24]	.18***	.05	[.08, .28]
*Mean*	.33***	.04	[.26, .40]	.33***	.04	[.26, .40]	.33***	.04	[.26, .40]	.33***	.04	[.26, .40]
*+1 SD*	.17***	.05	[.07, .27]	.48***	.05	[.38, .58]	.51***	.05	[.41, .61]	.48***	.05	[.38, .58]
**Negative emotions toward God**												
*Interaction*	-.03	.02	[-.08, .01]	-.01	.03	[-.05, .04]	-.01	.02	[-.06, .04]	-.05*	.03	[-.10, -.002]
*-1 SD*	-	-	-	-	-	-	-	-	-	.10*	.04	[.02, .19]
*Mean*	-	-	-	-	-	-	-	-	-	.04	.03	[-.02, .10]
*+1 SD*	-	-	-	-	-	-	-	-	-	-.03	.04	[-.11, .06]

Note

***p < .001

** *p* < .01

* *p* < .05 Emotional Stability not presented because it was not a statistically significant moderator.

#### Sample C (India): Differences between Hindu postures

**Fig 4:**
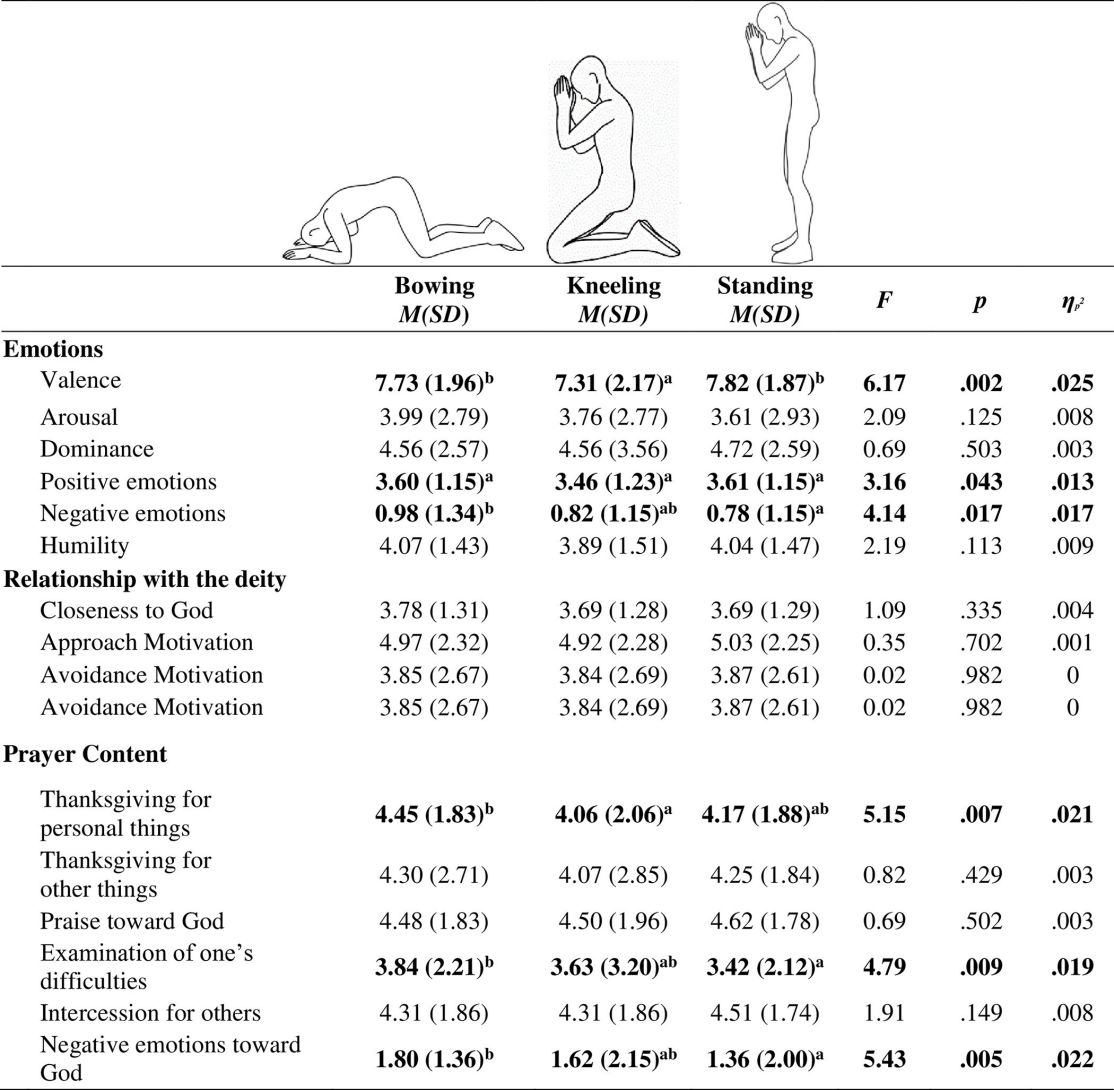
Means (standard deviations) of three postures (Sample C Hindus in India). *Note*. Main effects statistically significant at *p* < .05 are bolded. All *df*s = 2, 486–488. For each line, different letters subscripts indicate statistically significant differences for Sidak-corrected post-hoc tests.

Since participants did not complete questionnaires for postures that they found offensive to imagine praying in, sample size for the up/expansive postures was insufficient to run our usual comparison with the down/constrictive postures. Therefore, we chose post-hoc to only compare three common, well accepted Hindu postures (>50% reported personally using the posture), which were all varying degrees of down/constrictive: from somewhat, in *standing* with prayer hands; very, in *kneeling* with prayer hands; to extremely, in *bowing* while kneeling (see Fig 4).

We ran a one-way repeated measures ANOVA to compare these three Hindu postures (standing, kneeling, and bowing) on all outcomes followed by Sidak post-hoc comparisons. See Fig 4 for means, standard deviations, and results.

Surprisingly, participants reported significantly lower positive valence in kneeling posture than the other two, which did not differ from each other. As expected, participants reported the greatest negative emotion in the most down/constrictive posture, bowing, followed by kneeling, and then standing. Only the bowing and standing postures were significantly different from each other. There was no significant difference between postures on arousal, dominance, humility, or closeness to God.

There was a significant difference between postures on three prayer content themes: thanksgiving of personal things, examination of one’s difficulties, and negative emotions toward God. Examination of one’s difficulties was endorsed significantly more in bowing posture compared to standing, with kneeling falling in-between. The same pattern was found for negative emotions towards God. On thanksgiving of personal things, the bowing posture was again the highest, but followed by standing and then kneeling posture. Here, only bowing and kneeling postures were significantly different. There were no significant differences between the postures on thanksgiving for other things, praise towards God, or intercession for others.

## Discussion

Across the world and for millennia, people have communicated with their gods through postures and gestures [6]. Research on embodied processes in psychology supports the relevance of such physical expressions for religious experiences [3–6](_((((xxx. Yet, empirical data on the psychological implications of the gestures and postures that worshippers adopt during private and collective prayer are scarce. Given the frequency of bodily prayer postures among the estimated 6.12 billion religious-identifying people, we believe the present research is much needed.

We collected data among members of the three biggest religious traditions–Christianity, Islam, and Hinduism–across three countries. In order to test the causal effect of postures on experience, we developed a protocol asking respondents to imagine praying in various postures typically adopted in these religious traditions in order to trigger a multimodal network of stored experiences [12]. In a repeated-measures experimental research, after imagining praying in each posture, we collected information about emotions, relationship with deity, and prayer content.

Across religious groups in the U.S., and specifically focusing on Christian participants, results largely supported our hypotheses, aligning with previous research on the embodiment of affect (which also largely rests on Western Christian samples). Compared to downward and constrictive prayer postures, imagining praying in *upward and expansive* postures led to more positive emotions, arousal, and more prayers of praise (but surprisingly not thanksgiving), aligning with evidence that these postural features express and promote positive affect in everyday life [30] This postural family also led to more dominance, aligning with research suggesting that expansiveness also expresses and promotes high status, pride, and dominance in everyday life [13](_((((xxx. This last result may explain why some religious groups view upward and expansive postures as irreverent in a religious context. Together, these results depict upward and expansive prayer postures as supporting an uplifting, lively, and empowering religious experience.

In contrast, compared to upward and expansive postures, imagining praying in *downward and constrictive* postures led to more negative emotions (albeit still at very low levels), humility, and more prayers focused on examining one’s difficulties or making requests for others. For all groups except Christians, such postures also led to more perceived closeness to God. These results add to the broader picture of downward and constrictive postures triggering religiosity [42] and a religious mindset [43]. Together, these results depict downward and constrictive postures as supporting a religious experience characterized by humility and the search for closeness to God, which provides an avenue for both self-focused introspection and intercession on behalf of others. In sum, the diversity of postures observed in religious practices underscores the multifaceted nature of religious experiences and the many psychological and social functions these experiences fulfill.

Furthermore, we found significant nuances among the religious traditions, particularly regarding prayer content and the specific dimension of affective valence. In particular, the thanksgiving prayer theme was more elicited in upward and expansive postures among Christians, but less elicited in such postures among Muslims and U.S. Hindus (one exception for Turkish Muslims and thanksgiving for other things). In fact, among Turkish Muslims, all prayer contents were generally more elicited in downward and constrictive postures. In addition, Indian Hindus often felt discomfort with imagining upward and expansive prayer postures, leading to incomplete data for these positions. This reaction was unexpected, especially given similar traditional poses like Urdhva Hastasana. As suggested by the local third author, current political and interfaith sensitivities may cause hesitancy towards non-traditional practices, indicating that such postures may not be universally associated with positive feelings. As a result, we compared three downward and constrictive postures in this sample, but found small, if any, differences, and no clear pattern. Finally, Turkish Muslims and U.S. Hindus reported more approach AND avoidance motivation toward God in downward and constrictive postures, which may suggest that (a) the relationship with God is highly important in such postures, aligning with the finding that closeness to God is also increased, and (b) the particular motivations could not be well-distinguished by these measures or in these samples.

These results suggest that religious and country-level cultures imbue specific postures with distinct emotional and cognitive meanings through their unique experiences and interpretive systems (see for moderation by culture in the embodiment of other domains [44, 45]). These findings align with Cultural Schema Theory, which posits that cultures shape broad schemas–sets of cognitive associations that have developed over time and through repeated experiences [46, 47]). Grounded cognition further suggests that these schemas are multimodal, involving sensory inputs like visual, auditory, and kinesthetic elements, with bodily input having the capacity to trigger these schemas. In this research, we find that the specific multimodal associations (schemas) activated by imagined postures differ between religious cultures. This theory would also suggest that the religious context in which these postures are adopted may activate a different network of associations than if the same postures were adopted in a nonreligious context. Such comparison between contexts represents a fruitful area for future research.

Finally, we examined how individual differences might influence the psychological impact of prayer postures. Results showed no or very little evidence of moderation for religiosity and for the personality trait of emotional stability (no prior research had tested such moderation). In the U.S., results indicated more pronounced experiences among those less familiar with the postures and less engaged in regular religious practice. These results suggest that (a) less general experience with postures and practice may relate to more bodily input in the construction of the psychological experience, or that (b) more experience and practice is associated with less reactivity, potentially due to habituation. In contrast, those with higher extraversion showed less difference in response, suggesting a possible habituation to expansive postures (for work on expansiveness and extraversion, see [13]). People with higher agreeableness and conscientiousness, however, displayed greater differences, potentially due to increased engagement in the study procedure. Interoceptive sensibility did not significantly affect responses, marking a departure from previous findings in sensory embodiment research [48], and underscoring the need for further study in this emerging area of research.

We note a few limitations to this research. Overall, all effects were small and it is unclear if the effects are truly small or if the procedure of *imagining* praying in a posture rather than adopting it may have played a role. The study is also limited to the postures tested and organized into two broad postural families. For example, we cannot speak to idiosyncratic postures such as the lotus pose commonly adopted by Hindus, posture sequences (as in the Muslim salat), and movements and gestures beyond static postures. We further note that the choice of postures to be adopted in individual prayer and collective worship is regulated differently across and even within these religious traditions. For example, Muslims go through the same set of postures in sequence whereas many Christians and Hindus have more freedom in choosing their prayer postures. Finally, this study is limited to the samples tested here and we note in particular that we relied on Mturk respondents (Study 1) and that we observe an overrepresentation of female student participants in Studies 2–3. This is notable in the Muslim sample since males and females have different experiences of prayer. The general outline of prayer is the same but the embodiment of prayer can be slightly different between men and women depending on context and school of Islamic interpretation and they pray separately. In future research, such important nuances and potential differences could be addressed to increase the generalizability of the findings.

Understanding the psychological effects of prayer postures has implications for our understanding of how religious practices impact emotional experiences and potentially influence mental states. Research suggests that religious practices, including prayer, can have positive effects on mental health, such as reducing stress, anxiety, and promoting feelings of well-being and meaning in life. These effects are partially explained by experiences of positive emotions and closeness to a deity [49, 50]. The present research further positions physical body postures as one plausible mechanism by which benefits accrue over time. These findings may inform individuals and mental health practitioners on tailoring and incorporating religious practices for mental health and well-being, as well as those who are not affiliated with religion but engage in mind-body practices. Of particular relevance would be insight on how body posture influences emotional experience, potentially in application to mindfulness and mediation practices. As such, understanding the psychological effects of prayer postures has implications for well-being both within and outside of religious contexts.

## Supporting information

S1 File (DOCX)

## References

[1] Pew Research Center. The changing global religious landscape 2017 [Available from: https://www.pewresearch.org/religion/2017/04/05/the-changing-global-religious-landscape/.

[2] Pew Research Center. U.S. religious landscape study 2014 [Available from: https://www.pewresearch.org/religion/religious-landscape-study/frequency-of-prayer/.

[3] Barsalou LW, Barbey AK, Simmons WK, Santos A. Embodiment in religious knowledge. Journal of Cognition and Culture. 2005;5(1):14–57.

[4] Jones J. Living religion: Embodiment, theology, and the possibility of a spiritual sense. New York, NY: Oxford University Press; 2019.

[5] Soliman TM, Johnson KA, Song H. It’s not “all in your head” Understanding religion from an embodied cognition perspective. Perspectives on Psychological Science. 2015;10(6):852–64. doi: 10.1177/1745691615606373 26581739

[6] Van Cappellen P, Edwards ME. The embodiment of worship: Relations among postural, psychological, and physiological aspects of religious practice. Journal for the Cognitive Science of Religion. 2021;6(1–2):56–79.

[7] Barsalou LW. Grounded cognition. Annual Review of Psychology. 2008;59:617–45. doi: 10.1146/annurev.psych.59.103006.093639 17705682

[8] Meier BP, Schnall S, Schwarz N, Bargh JA. Embodiment in social psychology. Topics in cognitive science. 2012;4(4):705–16. doi: 10.1111/j.1756-8765.2012.01212.x 22777820

[9] Körner A, Topolinski S, Strack F. Routes to embodiment. Frontiers in Psychology. 2015;6. 26191033 10.3389/fpsyg.2015.00940 PMC4488596

[10] Jeannerod M. Neural simulation of action: a unifying mechanism for motor cognition. Neuroimage. 2001;14(1):S103–S9. 11373140 10.1006/nimg.2001.0832

[11] Loev S, Inchingolo M, Velasco PF. What is it (like) to imagine an emotion? Humanities and Social Sciences Communications. 2022;9(1):1–9.

[12] Gallese V. Embodied simulation: From neurons to phenomenal experience. Phenomenology and the Cognitive Sciences. 2005;4:23–48.

[13] Van Cappellen P, Edwards ME, Shiota MN. Shades of expansiveness: Postural expression of dominance, high-arousal positive affect, and warmth. Emotion. 2023;23(4):973–985. doi: 10.1037/emo0001146 36048034

[14] Mehrabian A, Russell JA. An approach to environmental psychology: The MIT Press; 1974.

[15] Fontaine JR, Scherer KR, Roesch EB, Ellsworth PC. The world of emotions is not two-dimensional. Psychological science. 2007;18(12):1050–7. doi: 10.1111/j.1467-9280.2007.02024.x 18031411

[16] Granqvist P, Mikulincer M, Shaver PR. Religion as attachment: Normative processes and individual differences. Personality and Social Psychology Review. 2010;14(1):49–59. doi: 10.1177/1088868309348618 20023208

[17] Corwin AI, Brown TW. Emotion in the language of prayer. In: Pritzker SE, Fenigsen J, Wilce JM, editors. The Routledge Handbook of Language and Emotion. New York, NY: Routledge; 2020. p. 325–43.

[18] Van Cappellen P, Cassidy S, Zhang R. Religion as an embodied practice: Organizing the various forms and documenting the meanings of Christian prayer postures. Psychology of Religion and Spirituality. 2023;15(2):251–261.

[19] Ladd KL, Spilka B. Inward, outward, upward prayer: Scale reliability and validation. Journal for the Scientific Study of Religion. 2006;45(2):233–51.

[20] Duclos SE, Laird JD, Schneider E, Sexter M, Stern L, Van Lighten O. Emotion-specific effects of facial expressions and postures on emotional experience. Journal of Personality and Social Psychology. 1989;57(1):100.

[21] Veenstra L, Schneider IK, Koole SL. Embodied mood regulation: The impact of body posture on mood recovery, negative thoughts, and mood-congruent recall. Cognition and Emotion. 2017;31(7):1361–76. doi: 10.1080/02699931.2016.1225003 27626675

[22] Riskind JH, Gotay CC. Physical posture: Could it have regulatory or feedback effects on motivation and emotion? Motivation and emotion. 1982;6(3):273–98.

[23] Ball G, Breese J. Relating personality and behavior: Posture and gestures. In: Paiva A, editor. Affective Interactions: Towards a new generation of computer interfaces: Springer; 2000. p. 196–203.

[24] D’Errico F, Poggi I. Tracking a leader’s humility and its emotions from body, face and voice. Web Intelligence. 2019;17(1):63–74.

[25] LaFrance M, Mayo CAW. Cultural Aspects of Nonverbal Communication. International Journal of Intercultural Relations. 1978;2:71–89.

[26] Wilkes C, Kydd R, Sagar M, Broadbent E. Upright posture improves affect and fatigue in people with depressive symptoms. Journal of Behavior Therapy and Experimental Psychiatry. 2017;54:143–9. doi: 10.1016/j.jbtep.2016.07.015 27494342

[27] Hall JA, Coats EJ, LeBeau LS. Nonverbal behavior and the vertical dimension of social relations: A meta-analysis. Psychological Bulletin. 2005;131(6):898–924. doi: 10.1037/0033-2909.131.6.898 16351328

[28] Gronau QF, Van Erp S, Heck DW, Cesario J, Jonas KJ, Wagenmakers E-J. A Bayesian model-averaged meta-analysis of the power pose effect with informed and default priors: the case of felt power. Comprehensive Results in Social Psychology. 2017;2(1):123–38.

[29] Tracy JL, Robins RW. Show your pride: Evidence for a discrete emotion expression. Psychological Science. 2004;15:194–7. doi: 10.1111/j.0956-7976.2004.01503008.x 15016291

[30] Witkower Z, Tracy JL. Bodily communication of emotion: Evidence for extrafacial behavioral expressions and available coding systems. Emotion Review. 2019;11(2):184–93.

[31] Van Cappellen P, Ladd KL, Cassidy S, Edwards ME, Fredrickson BL. Bodily feedback: expansive and upward posture facilitates the experience of positive affect. Cognition and Emotion. 2022;36(7):1327–42. doi: 10.1080/02699931.2022.2106945 35924432

[32] Van Cappellen P, Edwards ME. Emotion expression in context: Full body postures of Christian prayer orientations compared to secular emotions. J Nonverbal Behav. 2021;45:545–565.

[33] Smith H. The world’s religions. San Francisco: HarperOne; 1991.

[34] Mehling WE, Price C, Daubenmier JJ, Acree M, Bartmess E, Stewart A. The Multidimensional Assessment of Interoceptive Awareness (MAIA). PloS One. 2012;7(11):e48230. doi: 10.1371/journal.pone.0048230 23133619 PMC3486814

[35] Fredrickson BL, Tugade MM, Waugh CE, Larkin GR. What good are positive emotions in crisis? A prospective study of resilience and emotions following the terrorist attacks on the United States on September 11th, 2001. Journal of Personality and Social Psychology. 2003;84:365–76. 12585810 10.1037//0022-3514.84.2.365 PMC2755263

[36] Bradley MM, Lang PJ. Measuring emotion: The self-assessment manikin and the semantic differential. Journal of Behavior Therapy and Experimental Psychiatry. 1994;25(1):49–59. doi: 10.1016/0005-7916(94)90063-9 7962581

[37] Sharp CA, Johnson KA. Assessing spirituality on two dimensions: closeness to God and focal orientation. International Journal for the Psychology of Religion. 2020;30(1):48–67.

[38] Impett EA, Strachman A, Finkel EJ, Gable SL. Maintaining sexual desire in intimate relationships: The importance of approach goals. J Pers Soc Psychol. 2008;94(5):808–23. doi: 10.1037/0022-3514.94.5.808 18444740

[39] Idler EL, Musick MA, Ellison CG, George LK, Krause N, Ory MG, et al. Measuring multiple dimensions of religion and spirituality for health research conceptual background and findings from the 1998 general social survey. Research on Aging. 2003;25(4):327–65.

[40] Gosling SD, Rentfrow PJ, Swann WBJ. A very brief measure of the Big-Five personality domains. Journal of Research in Personality. 2003;37:504–28.

[41] Montoya AK, Hayes AF. Two-condition within-participant statistical mediation analysis: A path-analytic framework. Psychological Methods. 2017;22(1):6. doi: 10.1037/met0000086 27362267

[42] Fuller RC, Montgomery DE. Body posture and religious attitudes. Archive for the Psychology of Religion. 2015;37(3):227–39.

[43] Ransom MR, Alicke MD. On bended knee: Embodiment and religious judgments. Current Research in Social Psychology. 2013;21.

[44] Lee SW, Schwarz N. Bidirectionality, mediation, and moderation of metaphorical effects: The embodiment of social suspicion and fishy smells. Journal of personality and social psychology. 2012;103(5):737. doi: 10.1037/a0029708 22905770

[45] Park LE, Streamer L, Huang L, Galinsky AD. Stand tall, but don’t put your feet up: Universal and culturally-specific effects of expansive postures on power. Journal of Experimental Social Psychology. 2013;49(6):965–71.

[46] Hunzaker MF, Valentino L. Mapping cultural schemas: From theory to method. American Sociological Review. 2019;84(5):950–81.

[47] Boutyline A, Soter LK. Cultural schemas: What they are, how to find them, and what to do once you’ve caught one. American Sociological Review. 2021;86(4):728–58.

[48] Häfner M. When body and mind are talking. Experimental Psychology. 2013;60(4):255–9. 23548985 10.1027/1618-3169/a000194

[49] Van Cappellen P, Toth-Gauthier M, Saroglou V, Fredrickson BL. Religion and well-being: The mediating role of positive emotions. Journal of Happiness Studies. 2016;17(2):485–505.

[50] Hayward RD, Krause N. Religion, mental health, and well-being: Social aspects. In: Saroglou V, editor. Religion, Personality, and Social Behavior. New York, NY: Psychology Press; 2014. p. 255–80.

