# Supplementary material for: Kneel, stand, prostrate: The psychology of prayer postures in three world religions
Source: PLoS One. 2024 Aug 22;19(8):e0306924. doi: 10.1371/journal.pone.0306924 (PMC11341042; doi:10.1371/journal.pone.0306924)
Supplement: S1 File — (DOCX) [file pone.0306924.s001.docx]

Kneel, Stand, Prostrate: The Psychology of Prayer Postures in Three World Religions

Online Supplemental Materials

Table of Contents

[S1 Text. Discussion on Display Rules in Fig 1 1](#_Toc164778756)

[S2 Text. Exact statistics for Fig 2 data 4](#_Toc164778757)

[U.S. sample 4](#_Toc164778758)

[Muslims - Turkey 9](#_Toc164778759)

[S3 Text. Comparing U.S. Muslims and Turkish Muslims on Differences between Down/Constrictive and Up/Expansive Postures 10](#_Toc164778760)

[S1 Table. Experience with Postures Moderation in the Turkish Muslim Sample 12](#_Toc164778761)

# **S1 Text. Discussion on Display Rules in Fig 1**

If we take seriously the idea that postures impact the religious experience, then people may want to avoid imagining praying in certain postures or even find some postures offensive. To address this, we gave participants the option to indicate that a prayer posture was offensive to them and that they did not want to imagine praying in it. This step in the procedure incidentally provides us with interesting data on 1) whether we selected postures that people truly adopt in various faiths and 2) what are the display rules within each religion and culture. According to Ekman’s neurocultural theory of emotion (Ekman & Friesen, 1971), cultural display rules play a role in determining culture-specific differences in emotional displays. These rules are learned and culturally determined, and they govern how emotions are expressed based on social circumstances. Religions also likely shape cultural display rules for emotional expression. Therefore, we expect postures from a particular religious tradition to be more often adopted and less often perceived as offensive by members of that religious tradition compared to members of other religious traditions. For example, upward and expansive postures appear to be more central to Christian denominations and to Muslims (cf. standing up-right is one posture that is part of the salat, the traditional Muslim daily prayer ritual, and can be considered as upward and expansive).

Regarding the method, prior to each posture, participants selected if they (a) personally adopt this posture during prayer (b) do not adopt this posture during prayer, but are willing to *imagine* themselves in the posture, or (c) find this posture offensive and were not willing to imagine themselves praying in this posture. We present the percentages of each option for each posture, based on religious tradition, in Figure 1 in the main manuscript.

Results and Discussion: Regarding Christians, they reported adopting constrictive and downward Christian postures more frequently than expansive and upward Christian postures and overwhelmingly agreed to imagine themselves praying in every of these postures. These results align with self-reported postures adopted during a Christian Sunday service (Van Cappellen et al., 2023). Christians’ reports of finding postures offensive are generally low (<32%) but peak in the three specific non-Christian postures. Regarding Muslims, data from the Turkish Muslims clearly show that the majority (90-97%) report adopting the three Muslim postures. Surprisingly, a non-negligible number of U.S. Muslims (45-53% personally adopt and 26-28% find offensive) also report adopting non-Muslim postures, in stark contrast with Turkish Muslims (6-10% personally adopt and 67-70% find offensive). Similarly, among Hindus, the majority reports adopting the selected Hindu postures. But again, U.S. Hindus were more comfortable adopting non-Hindu postures (20-38% personally adopt and 28-47% find offensive) than Indian Hindus (2-3% personally adopt and 85-91% find offensive).

Together, these results suggest that the postures selected were indeed adopted by their respective tradition members. In addition, we observe that participants who belong to the majority religious group in their country - Muslims in Turkey, Hindus in India - compared to participants who belong to the religious minority in their country - Muslims and Hindus in the U.S. - report finding postures typical of the other religious groups more offensive. Many possible explanations exist for this difference.

First, the local intergroup contexts of Turkey and India may accentuate the importance of clearly communicating one’s religious identity as separate from other religious identities. For example, research by the Pew Research Center (2021) highlighted how much Indians value religious tolerance while also consistently preferring that religious communities live segregated lives. Given that prayer postures serve as visible signals of both self-identity (Ladd, 2007; Markus, 1977) and group membership, among other signals such as clothing, participants for whom signaling such membership as separate from others is particularly important may choose to not even engage with postures signaling other identities so as to avoid potential misidentification and to achieve self-identity consistency.

Second, beyond any self, intergroup, or social identity considerations, these participants may be more intuitively attuned to embodied processes and avoid any bad energy that they believe may come from imagining adopting a posture not typical of their faith practice. Finally, we cannot rule out a much more down to earth reason for the higher rates of postures found offensive in Turkey and India. Participants in these countries filled out paper questionnaires instead of online surveys, and may have seen more clearly that in selecting that a posture was offensive to them, they did not have to do the imagination exercise and thereby could avoid completing questions for such posture(s). Still, such skipping was not random nor generalized since they selectively skipped postures that were not typical of their tradition.

Finally, a non-negligible number of people found their own tradition postures offensive or reported adopting postures of other traditions. This suggests that the centrality of each posture to one’s faith tradition can vary by individual. We also recognize that the stylized depiction of posture may be slightly off (e.g., inclination of the head, position of prayer hands, degree of bow) for some participants. As a result, some of them may have in turn fully rejected the position. In future research, the study procedure could be altered for participants to first produce a list of postures they personally adopt, and then imagine praying in each one of them. The research team would need to organize the postures into similarly looking families post-hoc (see Van Cappellen et al., 2023 for method on how to ask for posture self-report by breaking it down to body parts)).

References

Ekman, P., & Friesen, W. V. (1971). Constants across cultures in the face and emotion. *Journal of Personality and Social Psychology*, *17*(2), 124-129.

Ladd, K. L. (2007). Religiosity, the need for structure, death attitudes, and funeral preferences. *Mental Health, Religion and Culture*, *10*(5), 451-472.

Markus, H. (1977). Self-schemata and processing information about the self. *Journal of Personality and Social Psychology*, *35*(2), 63-78.

Pew Research Center. (2021). *Religion in India: Tolerance and segregation*. <https://www.pewresearch.org/religion/2021/06/29/religion-in-india-tolerance-and-segregation/>

Van Cappellen, P., Cassidy, S., & Zhang, R. (2023). Religion as an embodied practice: Documenting the various forms, means, and associated experience of Christian prayer postures. *Psychology of Religion and Spirituality*, *15*, 251-261.

# **S2 Text. Exact statistics for Fig 2 data**

## U.S. sample

***Emotions***

**Overall Valence*.*** There was a significant interaction between religious tradition and posture for how much positive valence participants would feel in each posture, *F*(2, 1417) = 59.85, *p* < .001, η_p_^2^ = .078, therefore, we tested for simple main effects. Christians and Muslims reported significantly higher on positive valence in the up/expansive posture compared to the down/constrictive posture, respectively, *F*(1, 655) = 168.13, *p* < .001, η_p_^2^ = .204, and *F*(1, 455) = 7.00, *p* = .008, η_p_^2^ = .015. However, Hindus reported significantly lower on positive valence in the up/expansive posture compared to the down/constrictive posture, *F*(1, 307) = 5.76, *p* = .017, η_p_^2^ = .018.

**Arousal***.* There was a significant interaction between religious tradition and posture for how aroused participants would feel in each posture, *F*(2, 1417) = 35.08, *p* < .001, η_p_^2^ = .047. Christians and Hindus reported significantly higher arousal in the up/expansive postures than the down/constrictive postures, respectively, *F*(1, 655) = 194.70, *p* < .001, η_p_^2^ = .229 and *F*(1, 307) = 17.61, *p* < .001, η_p_^2^ = .054. Whereas, among Muslims, there was no significant difference between postures on arousal, *F*(1, 455) = 3.03, *p* = .083, η_p_^2^ = .007.

**Dominance***.* There was also a significant interaction between religious tradition and posture for how dominant participants would feel in each posture, *F*(2, 1417) = 17.22, *p* < .001, η_p_^2^ = .024. Christians, Muslims, and Hindus all reported that they would feel significantly more dominant in the up/expansive postures compared to the down/constrictive postures, respectively, *F*(1, 655) = 189.12, *p* < .001, η_p_^2^ = .224, *F*(1, 455) = 27.12, *p* < .001, η_p_^2^ = .056, and *F*(1, 307) = 32.42, *p* < .001, η_p_^2^ = .096. The significant interaction is due to the differences seen between religious groups. In this case, for both the up/expansive and down/constrictive postures, (respectively, *F*(1, 1421) = 31.70, *p* < .001, η_p_^2^ = .043, *F*(1, 1552) = 99.32, *p* < .001, η_p_^2^ = .113) Hindus and Muslims (who did not differ from each other) reported significantly greater dominance than Christians.

**Positive and Negative Emotions***.* For positive emotions, there was a significant interaction between religious tradition and posture, *F*(2, 1417) = 45.18, *p* < .001, η_p_^2^ = .060. Christians reported significantly greater positive emotions in the up/expansive postures compared to the down/constrictive postures, *F*(1, 655) = 85.66, *p* < .001, η_p_^2^ = .117. However, Hindus reported significantly lower positive emotions in the up/expansive postures compared to the down/constrictive postures, *F*(1, 307) = 15.69, *p* < .001, η_p_^2^ = .049. Finally, among Muslims there was no difference between posture dimensions, *F*(1, 455) = 0.099, *p* = .753, η_p_^2^ = 0.

For negative emotions, there was also a significant interaction between religious tradition and posture, *F*(2, 1417) = 39.55, *p* < .001, η_p_^2^ = .053. Again, Christians reported significantly lower negative emotions in the up/expansive postures compared to the down/constrictive postures, *F*(1, 655) = 116.31, *p* < .001, η_p_^2^ = .151. However, there was no differences between postural dimensions among Muslims, *F*(1, 455) = .50, *p* = .48, η_p_^2^ = .001, or Hindus, *F*(1, 307) = .73, *p* = .394, η_p_^2^ = .002.

**Humility***.* There was a significant interaction between religious tradition and posture on feeling humble, *F*(2, 1417) = 12.63, *p* < .001, η_p_^2^ = .018. Here, Christians, Muslims, and Hindus, all reported that they would feel significantly more humble in the down/constrictive postures compared to the up/expansive postures (respectively, *F*(1, 655) = 101.46, *p* < .001, η_p_^2^ = .134, *F*(1, 455) = 8.44, *p* = .004, η_p_^2^ = .018, and *F*(1, 307) = 38.71, *p* < .001, η_p_^2^ = .112). Similar to dominance, here the significant interaction is due to the differences seen between religious groups, where in the up/expansive postures, Muslims reported greater humility than Hindus, who, in turn, reported greater humility than Christians, *F*(1, 1421) = 16.65, *p* < .001, η_p_^2^ = .023, (no differences between religions were seen in the down/constrictive postures, *F*(1, 1552) = 1.38, *p* = .252, η_p_^2^ = .002).

***Relationship with God***

**Closeness to God***.* There was a significant interaction between religious tradition and posture on how close one feels to God in each posture, *F*(2, 1417) = 20.57, *p* < .001, η_p_^2^ = .028. Both Muslims and Hindus reported feeling significantly closer to God in the down/constrictive postures compared to the up/expansive postures, respectively, *F*(1, 455) = 11.68, *p* = .001, η_p_^2^ = .025 and *F*(1, 307) = 60.03, *p* < .001, η_p_^2^ = .164. Whereas there was no difference between postural dimensions for Christians in closeness to God, *F*(1, 655) = .301, *p* = .583, η_p_^2^ = 0.

**Approach and Avoidance Motivation toward God.** For approach motivation, there was a significant interaction between religious tradition and posture, *F*(2, 1417) = 12.95, *p* < .001, η_p_^2^ = .018. Christians and Muslims did not report differences in approach motivation between the two posture families, *F*(1, 655) = 0.74, *p* = .786, η_p_^2^ = .0. However, Hindus reported significantly lower approach motivation in the up/expansive postures compared to the down/constrictive postures, *F*(1, 307) = 41.54, *p* < .001, η_p_^2^ = .119.

For avoidance motivation, there was also a significant interaction between religious tradition and posture, *F*(2, 1417) = 6.93, *p* < .001, η_p_^2^ = .010. Christians reported significantly lower avoidance motivation in the up/expansive postures compared to the down/constrictive postures, *F*(1, 655) = 30.51, *p* < .001, η_p_^2^ = .045. However, Hindus demonstrated the opposite pattern, *F*(1, 307) = 16.13, *p* < .001, η_p_^2^ = .050. There was no difference between postural families among Muslims, *F*(1, 455) = .30, *p* = .586, η_p_^2^ = .001.

**Prayer Orientation***.* There was a significant interaction between religious tradition and posture for five out of the six prayer orientations: thanksgiving for personal things, *F*(2, 1417) = 26.63, *p* < .001, η_p_^2^ = .036, thanksgiving for other things, *F*(2, 1417) = 13.94, *p* < .001, η_p_^2^ = .019, praise toward God, *F*(2, 1417) = 44.28, *p* < .001, η_p_^2^ = .059, examination of one’s difficulties, *F*(2, 1417) = 50.76, *p* < .001, η_p_^2^ = .067, and intercession for others, *F*(2, 1417) = , *p* < .001, η_p_^2^ = .016. No interaction was found for negative emotions toward God, *F*(2, 1417) = 1.93, *p* = .146, η_p_^2^ = .003. Each of the significant interactions were followed up with tests for simple main effects within each religious tradition.

For the positive valence prayer themes, Christians reported significantly greater thanksgiving for personal things, *F*(1, 655) = 22.46, *p* < .001, η_p_^2^ = .033, thanksgiving for other things, *F*(1, 655) = 14.13, *p* < .001, η_p_^2^ = .021, and praise toward God, *F*(1, 655) = 95.85, *p* < .001, η_p_^2^ = .128, in the up/expansive postures than in the down/constrictive postures. However, Muslims and Hindus reported significantly less thanksgiving for personal things, (respectively, *F*(1, 455) = 5.78, *p* = .017, η_p_^2^ = .013, *F*(1, 307) = 28.08, *p* < .001, η_p_^2^ = .084), and thanksgiving for other things (respectively, *F*(1, 455) = 8.42, *p* = .004, η_p_^2^ = .018, *F*(1, 307) = 4.72, *p* = .031, η_p_^2^ = .015), in the up/expansive postures than in the down/constrictive postures. No differences were found between postures for praise towards God with Muslims, *F*(1, 455) = 1.60, *p* = .207, η_p_^2^ = .004, and only marginal differences found with Hindus, *F*(1, 307) = 3.86, *p* = .051, η_p_^2^ = .012).

For the negative valence prayer themes, Christians, Muslims, and Hindus all reported significantly greater examination of one’s difficulties in the down/constrictive postures compared to the up/expansive postures (respectively, *F*(1, 655) = 277.51, *p* < .001, η_p_^2^ = .298, *F*(1, 455) = 13.98, *p* < .001, η_p_^2^ = .030, and *F*(1, 307) = 27.18, *p* < .001, η_p_^2^ = .081). Similarly, Christians and Hindus reported significantly greater intercession for others in the down/constrictive postures, (respectively, *F*(1, 655) = 78.82, *p* < .001, η_p_^2^ = .107, *F*(1, 307) = 13.96, *p* < .001, η_p_^2^ = .043). Although, there was no significant difference for Muslims, *F*(1, 455) = 3.42, *p* = .065, η_p_^2^ = .007.

## Muslims - Turkey

***Emotions***

On the one hand, participants reported feeling significantly greater arousal, *F*(1, 335) = 8.81, *p* = .003, *η_p_^2^* = .026, and dominance, *F*(1, 332) = 62.95, *p* < .001, *η_p_^2^* = .159, in the up/expansive postures compared to the down/constrictive postures. On the other hand, participants reported greater negative emotions, *F*(1, 388) = 55.66, *p* < .001, *η_p_^2^* = .125, and humility, *F*(1, 400) = 22.89, *p* < .001, *η_p_^2^* = .054, in the down/constrictive postures compared to the up/expansive postures. We do not see a difference between groups in valence, *F*(1, 376) = .32, *p* = .571, *η_p_^2^* = .001 or positive emotions, *F*(1, 419) = 3.57, *p* = .060, *η_p_^2^* = .008.

***Relationship with God***

In the down/constrictive postures compared to the up/expansive postures, participants reported significantly greater closeness to God, *F*(1, 412) = 77.44, *p* < .001, *η_p_^2^* = .158, approach motivation toward God, *F*(1, 416) = 21.84, *p* < .001, *η_p_^2^* = .050, avoidance motivation toward God, *F*(1, 387) = 20.42, *p* < .001, *η_p_^2^* = .050, as well as all prayer orientations (examination of one’s difficulties, *F*(1, 407) = 96.69, *p* < .001, *η_p_^2^* = .192, intercession for others, *F*(1, 406) = 64.27, *p* < .001, *η_p_^2^* = .137, thanksgiving for personal things, *F*(1, 408 = 88.76, *p* < .001, *η_p_^2^* = .179, thanksgiving for other things, *F*(1, 403) = 53.69, *p* < .001, *η_p_^2^* = .118, and praise towards God, *F*(1, 417) = 50.71, *p* < .001, *η_p_^2^* = .108) with the exception of negative feelings towards God, where there were no differences between groups, *F*(1, 404) = .179, *p* = .672, *η_p_^2^* = 0.

# S3 Text. Comparing U.S. Muslims and Turkish Muslims on Differences between Down/Constrictive and Up/Expansive Postures

Among Muslim participants, we tested whether country (i.e., U.S. or Turkey) moderated the differences between the down/constrictive and up/expansive postures on experience using a mixed ANOVA with postural families as the within factor and country as the between factor. We found evidence for such interaction (see Figure below) showing that for 9 out of 14 outcomes, Muslims in Turkey reported more distinct experiences in the two postural families than Muslims in the U.S. In other words, the difference between the two postural families was often statistically significantly greater among Muslims in Turkey than Muslims in the U.S.

*
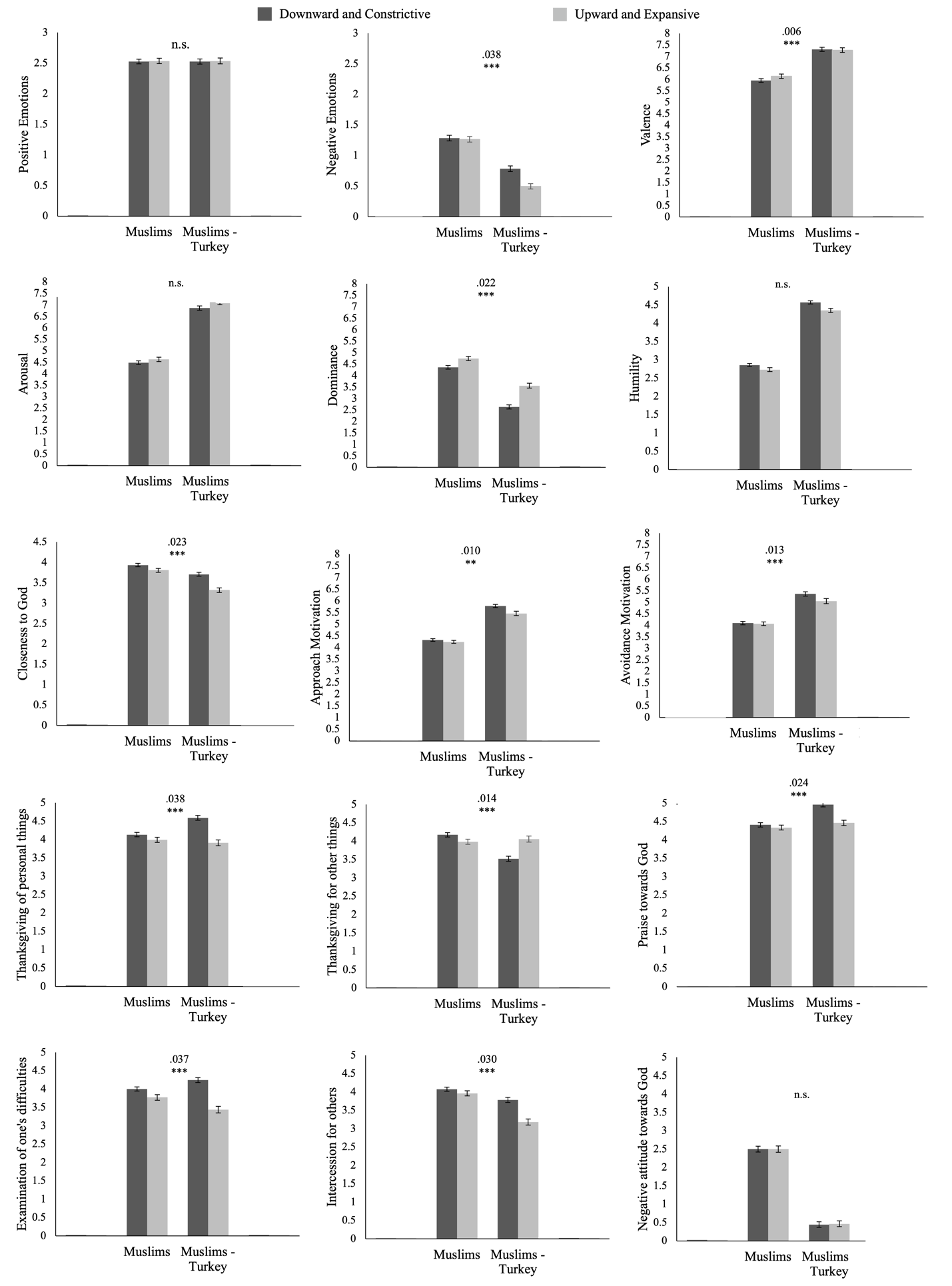
*

*Note.* “Muslims” come from Sample A, U.S. participants, and “Muslims – Turkey” come from Sample B, Turkish participants. Test statistics are provided for the interaction between country (between-subject factor) and postural families (withing-subject factor): ****p* < .001, ** *p* < .01, * *p* < .05, and number above refers to effect size *η_p_^2^*.

# S1 Table. Experience with Postures Moderation in the Turkish Muslim Sample

**Table S1**

*Moderation Tests for Difference between Down/Constrictive and Up/Expansive by Experience with Postures (Turkish Sample - Muslims)*

|  | ***B*** | ***SE*** | ***95% CI*** |
| --- | --- | --- | --- |
| **Emotions** |  |  |  |
| Positive Emotions |  |  |  |
| *Interaction* | -.17** | .06 | [-.29, -.05] |
| *-1 SD* | .18*** | .06 | [.07, .29] |
| *Mean* | .07 | .04 | [-.01, .14] |
| *+1 SD* | -.04 | .06 | [-.15, .07] |
| Negative Emotions |  |  |  |
| *Interaction* | -.19** | .06 | [-.31, -.07] |
| *-1 SD* | .43*** | .05 | [.32, .54] |
| *Mean* | .31*** | .04 | [.24, .39] |
| *+1 SD* | .19*** | .05 | [.09, .30] |
| Valence |  |  |  |
| *Interaction* | -.11 | .10 | [-.30, .09] |
| *-1 SD* | - | - | - |
| *Mean* | - | - | - |
| *+1 SD* | - | - | - |
| Arousal |  |  |  |
| *Interaction* | -.33** | .12 | [-.57, -.08] |
| *-1 SD* | -.04 | .11 | [-.26, .18] |
| *Mean* | -.25** | .08 | [-.41, -.09] |
| *+1 SD* | -.46*** | .11 | [-.69, -.24] |
| Humility |  |  |  |
| *Interaction* | -.40*** | .07 | [-.53, -.26] |
| *-1 SD* | .50*** | .06 | [.38, .62] |
| *Mean* | .25*** | .04 | [.16, .33] |
| *+1 SD* | -.004 | .06 | [-.13, .12] |
| Dominance |  |  |  |
| *Interaction* | -.08 | .18 | [-.42, .27] |
| *-1 SD* | - | - | - |
| *Mean* | - | - | - |
| *+1 SD* | - | - | - |
| **Relationship with God** |  |  |  |
| Closeness to God |  |  |  |
| *Interaction* | -.14** | .09 | [-.27, -.01] |
| *-1 SD* | .48*** | .06 | [.36, .60] |
| *Mean* | .39*** | .04 | [.30, .47] |
| *+1 SD* | .30*** | .06 | [.18, .42] |
| Approach Motivation |  |  |  |
| *Interaction* | -.29** | .11 | [-.51, -.07] |
| *-1 SD* | .50*** | .10 | [.31, .69] |
| *Mean* | .32*** | .07 | [.19, .46] |
| *+1 SD* | .15 | .10 | [-.04, .34] |
| Avoidance Motivation |  |  |  |
| *Interaction* | -.23* | .11 | [-.45, -.01] |
| *-1 SD* | .45*** | .10 | [.26, .65] |
| *Mean* | .31*** | .07 | [.18, .45] |
| *+1 SD* | .17 | .10 | [-.02, .36] |
| Examination of one’s difficulties |  |  |  |
| *Interaction* | -.16 | .12 | [-.41, .08] |
| *-1 SD* | - | - | - |
| *Mean* | - | - | - |
| *+1 SD* | - | - | - |
| Thanksgiving for personal things |  |  |  |
| *Interaction* | -.21* | .11 | [-.43, -.002] |
| *-1 SD* | .82*** | .10 | [.63, 1.01] |
| *Mean* | .68*** | .07 | [.55, .82] |
| *+1 SD* | .54*** | .10 | [.35, .74] |
| Intercession for others |  |  |  |
| *Interaction* | -.22* | .11 | [-.44, 0] |
| *-1 SD* | .74*** | .10 | [.54, .94] |
| *Mean* | .60*** | .07 | [.46, .74] |
| *+1 SD* | .46*** | .10 | [.26, .66] |
| Thanksgiving for other things |  |  |  |
| *Interaction* | -.26* | .12 | [-.49, -.04] |
| *-1 SD* | .71*** | .10 | [.51, .92] |
| *Mean* | .55*** | .07 | [.41, .69] |
| *+1 SD* | .38*** | .10 | [.18, .58] |
| Praise toward God |  |  |  |
| *Interaction* | -.27* | .11 | [-.48, -.05] |
| *-1 SD* | .65*** | .10 | [.46, .85] |
| *Mean* | .49*** | .07 | [.35, .63] |
| *+1 SD* | .32*** | .10 | [.13, .51] |
| Negative emotions toward God |  |  |  |
| *Interaction* | .05 | .08 | [-.11, .22] |
| *-1 SD* | - | - | - |
| *Mean* | - | - | - |
| *+1 SD* | - | - | - |

Note. **p* < .05, ***p* < .01, ****p* ≤ .001
